# Deep learning-based frame synthesis enables radiation dose reduction in digital subtraction angiography imaging: a multicenter study

**DOI:** 10.3389/fmed.2026.1793962

**Published:** 2026-05-15

**Authors:** Ruibo Liu, Ruixuan Zhang, Wei Qian, Guobiao Liang, Guangxin Chu, Yuwei Han, Xiaochuan Xu, Hai Jin, Ligang Chen, Jing Li, He Ma

**Affiliations:** 1 College of Medicine and Biological Information Engineering, Northeastern University, Shenyang, China; 2 Department of Neurosurgery, General Hospital of Northern Theater Command, Shenyang, China; 3 Neurointerventional Department, 242 Hospital Affiliated to Shenyang Medical College, Shenyang, China; 4 Department of Neurology, The Fourth Affiliated Hospital of China Medical University, Shenyang, China

**Keywords:** deep learning, digital subtraction angiography, image quality assessment, low-dose radiation, multicenter validation, video frame generation

## Abstract

**Background:**

As an important imaging tool for diagnosing and treating cerebrovascular diseases, the low-dose imaging technology of digital subtraction angiography (DSA) can effectively reduce radiation exposure risks for both patients and operators. To ensure clinical demand while minimizing radiation dose, this study proposed SAVE-Net, which integrates deep learning with optical flow estimation. The model is designed to synthesize intermediate frames in DSA sequences, thereby reducing the number of scans required in clinical practice and directly decreasing the radiation dose.

**Methods:**

SAVE-Net was developed to generate subsequent frames following any given real DSA frame. A total of 17,335 DSA sequences from one hospital were used for model training, fine-tuning, and internal validation. For external validation, an additional 3,255 DSA sequences from two other hospitals were utilized. Image similarity between generated and real frames was quantitatively evaluated using the Structural Similarity Index (SSIM) and Peak Signal-to-Noise Ratio (PSNR). Furthermore, five interventional radiologists independently performed a visual Turing test and quality assessment on the generated sequences. Inter-rater agreement for the Turing test results was assessed using Fleiss' Kappa, while the Wilcoxon Signed-Rank test was employed to analyze significant differences in the quality ratings.

**Results:**

Internal validation results demonstrated that SAVE-Net achieved DSA sequences highly consistent with real clinical data using only 1/7 of the standard radiation dose and maintained stable performance across multiple scenarios. External validation results illustrated that SAVE-Net achieved an effective performance [SSIM: 0.951, 95% CI: (0.948, 0.956); PSNR: 40.764, 95% CI: (40.673, 40.798); and generation time: 0.04 s/frame]. Assessment results showed no significant difference between the generated sequences and the real ones. Additionally, the generated results exhibited high consistency with real data in terms of overall image quality (4.919 vs. 4.940) and diagnostic confidence (4.838 vs. 4.910).

**Conclusion:**

SAVE-Net enables the generation of clinically diagnostic DSA sequences using only 1/7 of the standard radiation dose, with image quality and diagnostic confidence comparable to real clinical data. Its superior performance across multi-center validation demonstrates a practical and effective approach to reducing radiation exposure in cerebrovascular imaging.

## 1 Introduction

Digital subtraction angiography (DSA) is the gold standard imaging modality for the diagnosis and treatment of vascular diseases, including cerebrovascular, cardiovascular, and peripheral vascular diseases (1–4). With the rapid advancement of interventional radiology, DSA has become indispensable in diverse clinical applications, such as vascular malformation correction, aneurysm management, and arterial stenosis evaluation (5–8).

However, conventional DSA protocols require multiple sequential acquisitions, leading to considerable radiation exposure for both patients and operators. Complex interventional procedures often entail high cumulative doses, elevating the risk of deterministic (e.g., skin injury) and stochastic (e.g., carcinogenesis) effects (9, 10). The intrinsic trade-off between the radiation dose and image quality further complicates clinical practice. Although techniques such as field-of-view (FOV) reduction can improve spatial resolution, they simultaneously increase the incident air kerma rate at the patient's surface (11). Conventional dose-reduction strategies, such as adjusting the tube voltage and current-time product, are inherently constrained by diagnostic image quality requirements, often failing to achieve substantial dose reduction without compromising clinical utility (12). Arteriovenous malformations (AVMs) and arteriovenous fistulas (AVFs), which are types of arteriovenous shunts (AV-shunts), are especially challenging to see on imaging because their small shunt structures are only visible for 1–2 frames during contrast passage. This makes DSA imaging very demanding in terms of spatial resolution and temporal fidelity.

Recent efforts to mitigate radiation exposure have explored hardware and software innovations. The adoption of digital flat-panel detectors has improved dose utilization efficiency (13–15), whereas digital variance angiography employs advanced algorithms to enhance vascular visualization under reduced per-frame dose. On the software side, digital variance angiography employs a unique variance algorithm to process raw image sequences, enabling enhanced vascular visualization even with reduced per-frame radiation dose (16–18). Nonetheless, these approaches remain limited by physical and practical limitations and are often unable to achieve significant dose reduction without degrading diagnostic fidelity, particularly in complex cases where high image quality is crucial for accurate diagnosis.

In contrast, artificial intelligence, particularly deep learning, offers a promising paradigm for transcending these constraints. Generative models, including generative adversarial networks (GANs), diffusion models, and neural radiance fields (NeRF), have demonstrated their potential in synthesizing high-quality DSA images from sparse data, effectively suppressing noise and artifacts (19–22). Emerging studies suggest that intermediate views can be synthesized from as few as two endpoint projections, potentially reducing radiation exposure to 1/3 of the conventional levels (23, 24). This points toward a new ultralow-dose DSA framework: acquiring sparse projections and reconstructing intermediate views algorithmically, thereby preserving image quality while markedly lowering the radiation dose.

Despite these advances, existing deep learning methods often achieve only modest dose reductions and frequently suffer from inter-frame discontinuity, limiting their clinical applicability. To address these challenges, we propose Sparse-view Angiographic Video Estimation Network (SAVE-Net), an end-to-end generative system for ultra-low-dose DSA imaging. SAVE-Net integrates convolution and transformer architectures with direction-aware feature extraction and bidirectional optical flow estimation, effectively modeling complex vascular motion to generate temporally coherent and anatomically faithful sequences. SAVE-Net can reduce radiation dose by up to 85.71% while maintaining clinically acceptable image quality. Additionally, the system provides a real-time inference speed of 0.04 s per frame, which ensures it meets the latency requirements for smooth interventional navigation. Through extensive multi-center validation, the framework exhibits robust generalizability and demonstrates strong potential for direct clinical translation. In summary, this study presents a viable solution for interventional radiology, integrating enhanced patient safety with maintained diagnostic efficacy.

## 2 Materials and methods

### 2.1 Patients

This study utilized DSA sequences collected from three independent hospitals to develop and evaluate SAVE-Net for multi-frame synthesis. The inclusion and exclusion criteria and data division details are shown in Supplementary Figure S1. Sequences were classified into two types based on scanning modes:

- 2D-DSA: A planar imaging modality acquired at a fixed C-arm position. It employs background subtraction to highlight temporal contrast dynamics within vessels, thereby providing real-time hemodynamic guidance during interventional procedures.
- 3D-DSA: Images acquired during a continuous 180° rotation of the C-arm around the patient, which is the gold standard for preoperative anatomical assessment of complex cerebrovascular lesions. Unlike 2D-DSA, 3D rotational DSA sequences have two core characteristics: (1) frame-to-frame changes include not only the temporal dynamic of contrast media filling, but also continuous spatial perspective changes, vascular projection deformation, and dynamic variation of vascular overlap caused by C-arm rotation; (2) 3D-DSA requires continuous acquisition during rotation, resulting in higher cumulative radiation exposure compared to 2D-DSA, which makes it a more critical scenario for low-dose imaging optimization.

### 2.2 Data preprocessing

**Figure 1:**
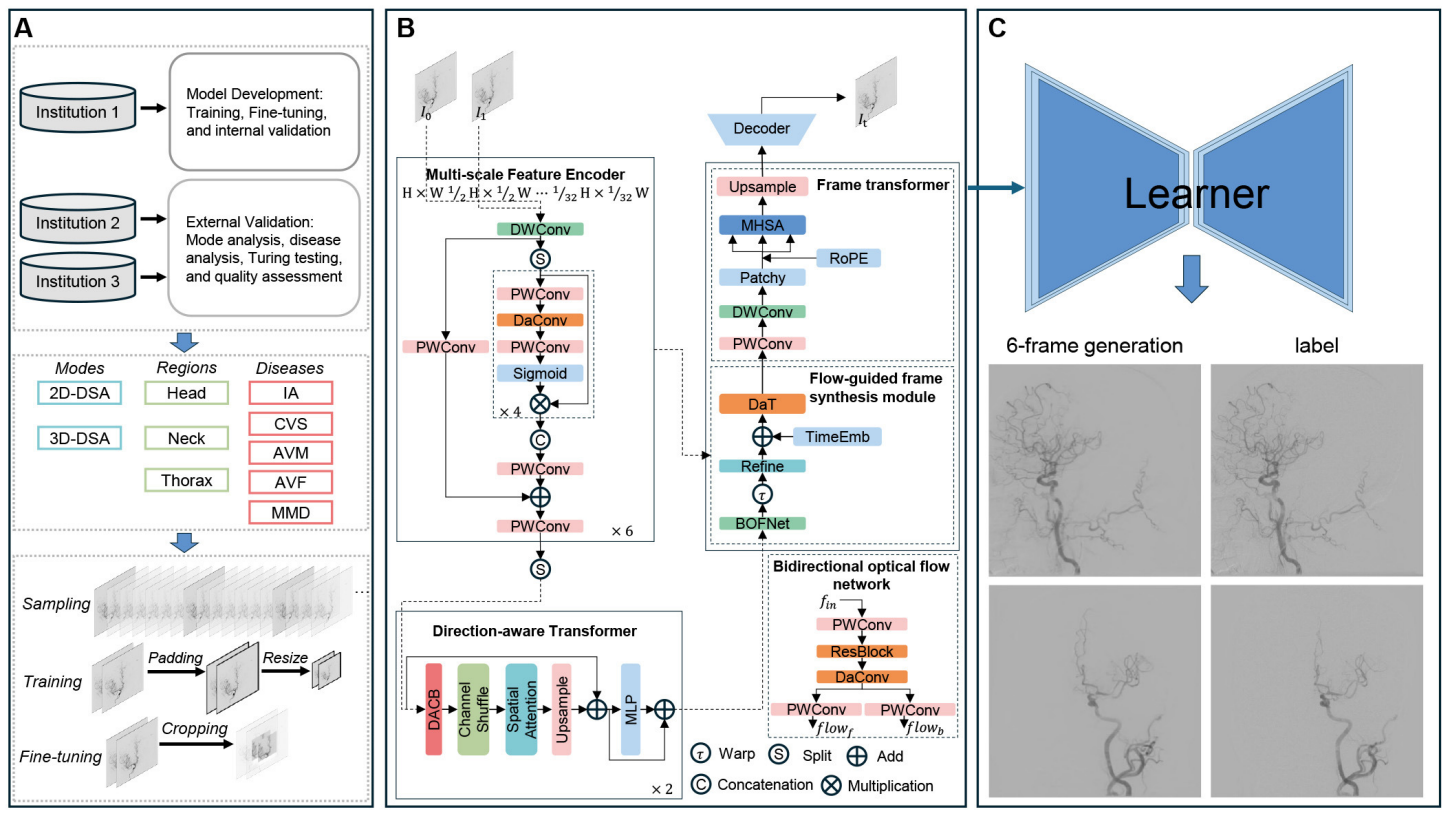
Overview of the pipeline and network architecture. **(A)** The dataset from three independent institutions, containing 2 different scanning modes, 3 distinct anatomical regions, and 5 disease types, was processed through sampling and cropping to serve as training data for the network. **(B)** Overall network pipeline of SAVE-Net. **(C)** The trained network's prediction result images (frontal and lateral views) for a random sample, along with the corresponding ground truth images.

All DSA sequences exhibited variable spatial resolutions, including but not limited to: 480 × 480, 512 × 512, 720 × 720, 960 × 960, 960 × 1, 240, 1, 024 × 1, 024, and 1, 440 × 1, 440 pixels. A standardized preprocessing pipeline was implemented to ensure uniform input dimensions, as shown in Figure 1A. During model training, images were padded into squares and resized to 128 × 128 to accelerate computation. This approach fully preserves the original morphology, proportions, and spatial relationships of the blood vessels. For fine-tuning, images were cropped with a uniform size of 512 × 512. The model has already learned basic vascular motion and structural features at low resolutions. Cropping allows the model to focus on regions containing major cerebral vascular structures while retaining sufficient spatial resolution to capture fine vascular details. Intensity values were normalized to the range [−1, 1] via min-max normalization.

### 2.3 Model architecture

The pipeline of the SAVE-Net is outlined in Figure 1B. The model incorporated a direction-aware feature extraction mechanism and an optical flow-based frame synthesis module. The network comprised four core components: the Multi-scale Feature Encoder (MFE), the Flow-Guided Frame Synthesis (FGFS) module, the Spatio-Temporal Fusion Bottleneck (STFB), and the Multi-scale Feature Decoder (MFD). Both the MFE and MFD employed a novel Direction-Aware Convolution Block (DACB) to enhance sensitivity toward vascular structures. The FGFS module combined a bidirectional optical flow estimation network with a feature refinement sub-network, enabling the synthesis of intermediate frame features across multiple scales. Within the STFB, we first applied direction-aware global attention to construct intra-frame representations. The FGFS module then synthesized the intermediate frame features, after which we leveraged inter-frame global attention to enhance temporal consistency and feature alignment (Supplementary material 1).

For 3D rotational DSA sequences with continuous perspective changes and vascular projection deformation, the model was specifically optimized in three core modules to ensure stable reconstruction performance: (1) The Direction-Aware Convolution Block (DACB) in the multi-scale encoder and decoder can adapt to the dynamic changes of vascular projection direction caused by C-arm rotation, maintaining high sensitivity to tiny vascular structures under different viewing angles; (2) The bidirectional optical flow estimation network in the Flow-Guided Frame Synthesis (FGFS) module can simultaneously model the temporal motion of contrast media filling and the spatial displacement of vascular structures caused by C-arm rotation, ensuring the spatial consistency of generated frames under different perspectives; (3) The Frame Transformer (FT) in the Spatio-Temporal Fusion Bottleneck (STFB) captures the global spatial correspondence of vascular structures across frames with different viewing angles via inter-frame global attention, solving the problem of dynamic vascular overlap in 3D rotational sequences and ensuring the anatomical fidelity of generated frames.

A loss function was employed, imposing constraints from both pixel space and feature space (Supplementary material 2). In the pixel space, the L1 loss function and SSIM loss function were applied, whereas features extracted from multiple layers of a pretrained ResNet34 network were used to compute L1 loss and MSE loss. We employed a two-phase training strategy for the loss weights. During the initial 50 epochs, we set (α, β, γ, δ) = (1, 1, 0, 0) to establish a strong baseline via pixel-level and structural constraints. For the remaining epochs, we adjusted the weights to (0.5, 0.7, 40, 40) to enhance semantic and stylistic consistency.

We configured the network with progressively increasing channel dimensions with the initial number being 16 and twice the previous layer. We defined the timestep as a uniform variable in the range [−1, 1], assigning –1 to the first frame and 1 to the last frame. We optimized the model using the AdamW optimizer with an initial learning rate of 0.0001 and applied a cosine annealing schedule with a 50-epoch period to gradually reduce the learning rate to a minimum of 1 × 10^−18^. The total training duration was 1,000 epochs. All experiments were conducted on an NVIDIA RTX A6000 (48GB) GPU, with a batch size of 8 for generating 1–6 frames and 4 for generating 7–8 frames. Our framework is trained and implemented in PyTorch 2.5.1 (25). The python version is 3.11.13. All statistical analyses were performed using the scikit-image package (26) version 0.25.2.

### 2.4 Statistical analysis

The generative capability of the proposed model was evaluated using the Structural Similarity Index (SSIM), Peak Signal-to-Noise Ratio (PSNR), Mean Square Error (MSE), and generation time. The prediction sequences for generating 1 to 8 frames were sequentially assessed and subjected to Turing tests to determine the maximum acceptable number of generation frames and the consistency with real sequences. The performance of the generation results was evaluated across multiple scenarios. Consistency between the generation results and the real sequences was evaluated in two dimensions: overall quality assessment and diagnostic confidence assessment.

For continuous variables, the paired t-test or the Mann-Whitney U test was applied; categorical variables were compared using the chi-square test; and the Kruskal-Wallis test was employed for analysis of ordinal variables. Data analysis was performed using Python (version 3.11). A *P* < 0.05 was considered statistically significant.

Although no formal diagnostic consistency metrics were prospectively defined, the study indirectly assessed diagnostic consistency through several complementary approaches. First, diagnostic confidence scores were independently rated by five interventional radiologists across all disease subgroups. Second, subgroup-specific quantitative metrics and time-intensity curve analysis were used to evaluate the fidelity of hemodynamic and structural features essential for lesion detection and grading. Third, guidewire tip displacement error was measured to assess spatial precision relevant to interventional navigation. These indirect indicators collectively support the diagnostic equivalence between real and generated sequences, as further detailed in the Results and Discussion sections.

## 3 Results

### 3.1 Patient characteristics

The baseline data for all included patients are summarized in Table 1. Dataset 1 consisted of 17,335 DSA sequences obtained from 15,286 patients, who underwent cerebral DSA examinations at a single institution using the SIEMENS AXIOM Artis system. This dataset was divided into training, fine-tuning, and internal test subsets for model development and initial evaluation. Dataset 2 included 3,255 DSA sequences collected from two external institutions using GE MEDICAL SYSTEMS and Philips Azurion 5M20. This dataset was used exclusively for external evaluation to assess the generalizability of the proposed model across different scanner manufacturers. To analyze the performance of our model in different situations, we categorized the data into 11 groups based on scanning modes (2D-DSA and 3D-DSA), two body parts (head and thorax), and cerebrovascular disease types (intracranial aneurysm [IA], cerebrovascular stenosis [CVS], AVM, AVF, and moyamoya disease [MMD]). Among them, AVM and AVF are collectively referred to as AV-shunts, which are characterized by direct arteriovenous blood flow bypassing the capillary bed, often with tiny shunt structures that are only transiently visible during DSA acquisition.

**Table 1:** Baseline information of our datasets.

Characteristics	3D			2D	
	Training	Fine-tuning	Testing	Fine-tuning	Testing
Dataset 1: 17,335 DSA sequences					
Age, year (IQR)	55 (46–63)	56 (42–65)	54 (41–71)	55 (38–69)	57 (43–74)
Gender					
Male, *n* (%)	1,376 (59.45)	715 (61.81)	223 (57.89)	4,941 (54.05)	1,225 (53.57)
Female, *n* (%)	938 (40.55)	442 (38.19)	163 (42.11)	4,202 (45.95)	1,061 (46.43)
Body parts					
Head and neck	2,314	1,157	386	8,244	1,783
Thorax	0	0	0	2,695	952
Diseases (available)					
Intracranial aneurysms	874	453	131	3,215	754
Cerebral vascular stenosis	732	353	112	2,876	496
Arteriovenous malformation	365	158	62	893	320
Arteriovenous fistula	186	152	58	762	102
Moyamoya	103	32	12	364	97
Dataset 2: 3,255 DSA sequences					
Body parts					
Head and neck	-	-	679	-	2,180
Thorax	-	-	0	-	396
Diseases (available)					
Intracranial aneurysm (IA)	-	-	310	-	848
Cerebral vascular stenosis (CVS)	-	-	183	-	685
Arteriovenous malformation (AVM)	-	-	81	-	359
Arteriovenous fistula (AVF)	-	-	65	-	203
Moyamoya disease (MMD)	-	-	37	-	78

### 3.2 Working mechanism of SAVE-Net

The radiation dose in DSA exhibits a strictly linear positive correlation with the actual number of frames acquired under identical tube voltage, tube current, and exposure parameters. This is a fundamental physical principle of X-ray imaging and the standard method for dose calculation in this field, consistent with the computational logic used in similar studies. In this study, the dose reduction logic of SAVE-Net is as follows: traditional DSA requires the continuous acquisition of every single image frame, and the total dose is proportional to the total number of acquired frames; SAVE-Net, however, employs a sparse acquisition strategy: for every 1 frame of real-world imagery acquired, the model generates N interpolated frames. Consequently, the total number of acquired frames is only 1/(N+1) of that in the traditional protocol, and the radiation dose is correspondingly reduced to 1/(N+1) of the original value.

During the acquisition process, the DSA device scans according to the sparse acquisition protocol, which involves capturing one real image every N frames (corresponding to the generation of N intermediate frames). For example, under the optimal 6-frame generation configuration, the device captures a real image once every 7 frames. During the generation phase, any two real-world acquired frames with specified frame intervals are input into SAVE-Net, and the model automatically generates all consecutive intermediate frames between these two frames. During the output phase, the generated frames are concatenated in chronological order to produce a complete DSA sequence that is exactly consistent in frame count and temporal resolution with the traditional full-sampling protocol.

### 3.3 Evaluation of frame generation capability

**Figure 2:**
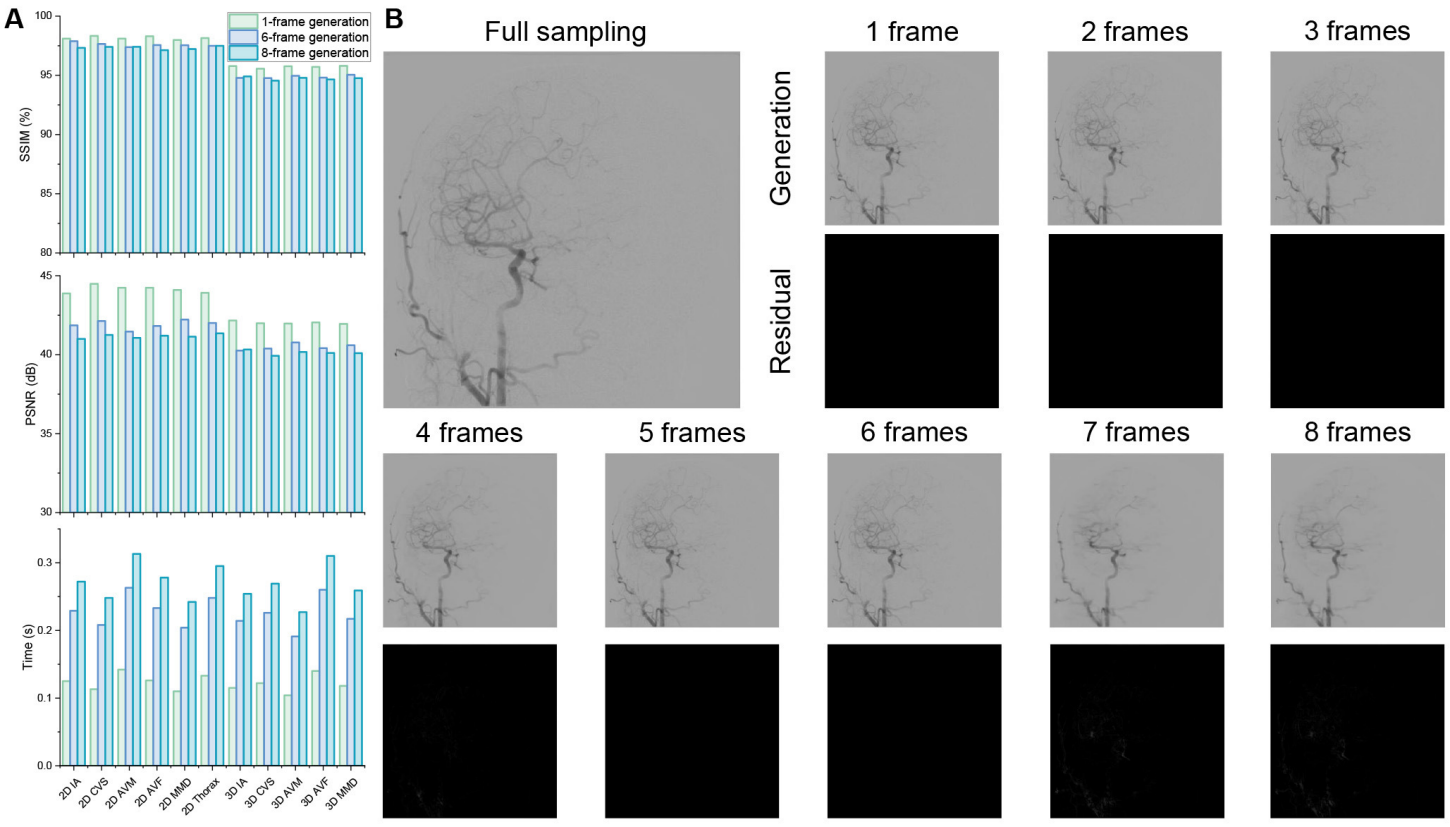
Performance analysis of SAVE-Net with varying numbers of generated frames. **(A)** Comparisons of SSIM, PSNR, and generation time across different scanning modes, anatomical regions, and disease types under 1-, 6-, and 8-frame generation conditions. **(B)** 1- to 8-frame generation results and the associated residual images.

To systematically evaluate the frame-generation performance of SAVE-Net, the optimal number of generated frames was investigated using sequences where 1 to 8 frames were synthesized after each captured real frame in Dataset 1. The implementation of this strategy achieves a radiation dose reduction to 1/2–1/9 of the conventional dose. The overall results shown in Figure 2 are illustrated in Supplementary Table S3. According to the experimental results, both SSIM and PSNR remained excellent across 1- to 8-frame generation, with no significant increase in generation time. For all categories, SSIM values ranged from 0.954 [95% CI: (0.953, 0.955)] to 0.964 [95% CI: (0.962, 0.965)], while PSNR values ranged from 40.391 dB [95% CI: (40.270, 40.513)] to 42.568 dB [95% CI: (42.397, 42.739)]. The corresponding generation time varied between 0.123 s and 0.270 s. These results indicate that SAVE-Net maintains strong performance across different anatomical regions and disease types. Figure 2B shows a lateral view frame from a randomly selected 3D head data along with the corresponding generated outputs for different generated frame numbers, which demonstrates high consistency across all outputs. Furthermore, residual maps reveal no visually detectable residual signals for 1- to 6-frame generation, and only minimal residual signals for 7- and 8-frame generation. For 3D-DSA sequences, the model also maintained stable performance across 1- to 6-frame generation, with SSIM values ranging from 0.947 to 0.957 and PSNR values consistently above 40 dB. Consistent with 2D-DSA, 6-frame generation was identified as the optimal clinical configuration for 3D-DSA, with no visually detectable residual signals in the residual maps.

**Figure 3:**
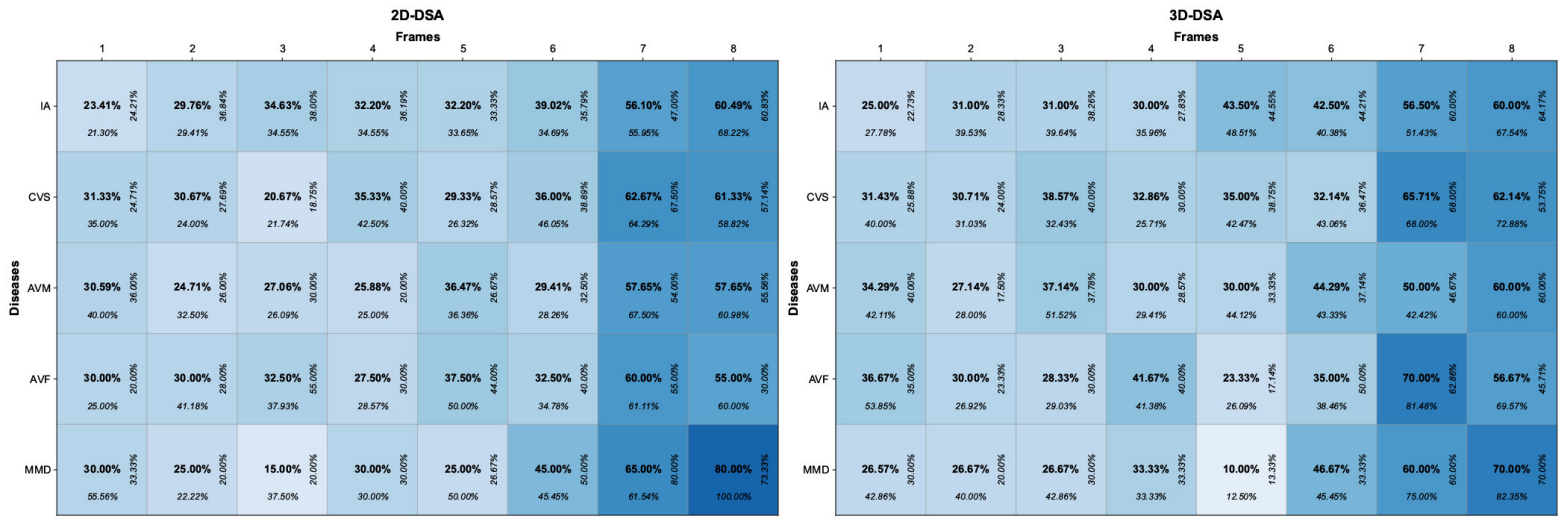
Confusion matrices of discriminating actual 2D- and 3D-DSA sequences from generated sequences. The numbers in the matrix represent accuracy, sensitivity, and recall respectively.

To further determine the optimal number of frames that SAVE-Net can generate while meeting clinical requirements, five interventional radiologists with 10-15 years of experience evaluated 200 randomly sampled sequence pairs (1–8 frame generation results and the corresponding real sequence) to judge whether each was real or synthetic. The 200 pairs consisted of: 41 pairs of 2D-IA, 40 pairs of 3D-IA, 30 pairs of 2D-CVS, 28 pairs of 3D-CVS, 17 pairs of 2D-AVM, 14 pairs of 3D-AVM, 8 pairs of 2D-AVF, 12 pairs of 3D-AVF, 4 pairs of 2D-MMD, and 6 pairs of 3D-MMD images. For each assessment round, the sequences were randomly ordered, and either a real sequence or a generated output was randomly selected. The radiologists were asked to determine the source of each sample, and their responses were recorded (Supplementary Data 1). Based on the confusion matrices and consistency analysis for 2D and 3D sequences (Figure 3 and Supplementary Table S4), all five radiologists were unable to reliably distinguish between real and generated sequences for 1- to 6-frame results (k values range from 0.020 to 0.126, all *P* < 0.05). In contrast, the ability to differentiate real from generated sequences improved obviously for 7- and 8-frame results. Moreover, radiologists were unable to reliably distinguish between real and generated 3D sequences in the visual Turing test (Fleiss' Kappa = 0.108, *P* < 0.05).

These results indicate that 1- to 6-frame results are suitable for practical clinical applications, whereas 7- and 8-frame results show significant differences from real sequences. Consequently, 6-frame generation was identified as the optimal clinical configuration, requiring only 1/7 of the radiation dose needed in conventional clinical practice.

### 3.4 Quantitative evaluation of generated DSA sequences

SAVE-Net was further evaluated on the external test set (Dataset 2). GenDSA (23) and GaraMoSt (24) were utilized to compare with SAVE-Net.

**Figure 4:**
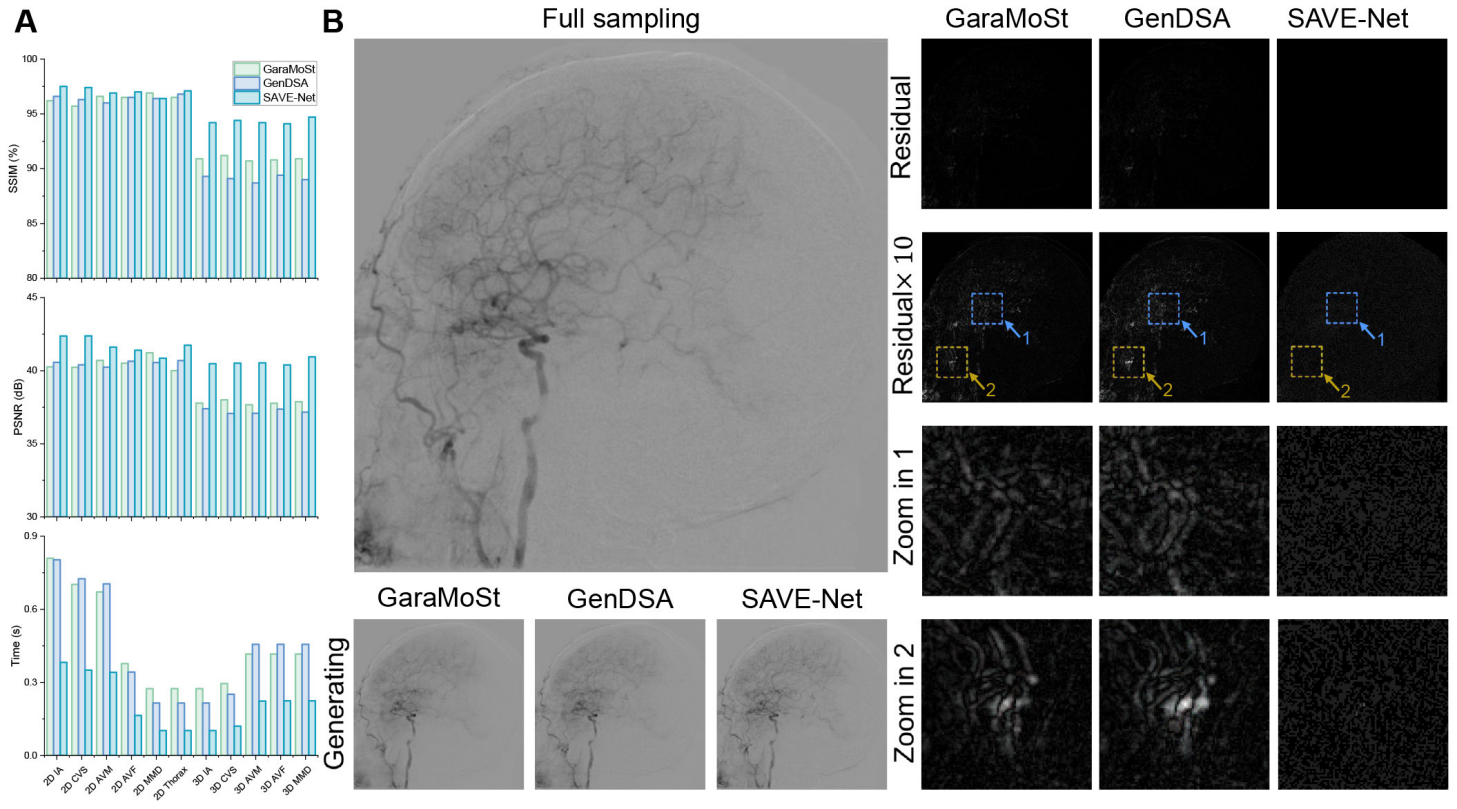
Comparative analysis of the 6-frame generation results. **(A)** Comparisons of SSIM, PSNR, and time for the results of the 6-frame generation among GaraMoSt, GenDSA, and SAVE-Net. **(B)** Comparisons of the generated images and residual images based on the 6-frame generation results by using GaraMoSt, GenDSA, and SAVE-Net.

Quantitative results for SSIM, PSNR, and generation time are summarized in Figure 4A and Supplementary Table S5. The results demonstrated that SAVE-Net outperformed both MoStNet and GaraMoSt across all metrics. Specifically, SAVE-Net achieved an average SSIM of 0.951 [95% CI: (0.948, 0.956)] and a PSNR of 40.764 dB [95% CI: (40.673, 40.798)], significantly higher than GenDSA {SSIM: 0.915 [95% CI: (0.914, 0.916)]; PSNR: 38.313 dB [95% CI: (38.252, 38.374)])} and GaraMoSt (SSIM: 0.927 [95% CI: (0.926, 0.928)]; PSNR: 38.705 dB [95% CI: (38.644, 38.766)]). In the 3D-DSA subgroup, SAVE-Net also significantly outperformed the state-of-the-art GenDSA and GaraMoSt models, achieving an average SSIM of 0.943 and PSNR of 40.573 dB, which were 5.8% and 9.0% higher than GenDSA and 3.7% and 7.3% higher than GaraMoSt, respectively.

A visual comparison based on a random 3D head keyframe is shown in Figure 4B, where SAVE-Net-generated images exhibit clearer vascular structures compared to the other two models. The residual maps indicate that the results generated by GenDSA contain more conspicuous artifacts. At 10 × zoom and enhancement, both GenDSA and GaraMoSt exhibit perceptible vascular contours. Additionally, SAVE-Net demonstrated superior computational efficiency with a generation time of only 0.212 s on average, which is approximately 2.1 times faster than both GaraMoSt (0.447 s) and GenDSA (0.440 s). This rapid processing capability highlights SAVE-Net's suitability for real-time imaging, effectively meeting the critical demands of interventional procedures. Meanwhile, the average generation time per frame for 3D sequences was only 0.180 s, which fully meets the real-time requirements of clinical 3D-DSA acquisition.

### 3.5 Evaluation of overall image quality and diagnostic confidence

**Figure 5:**
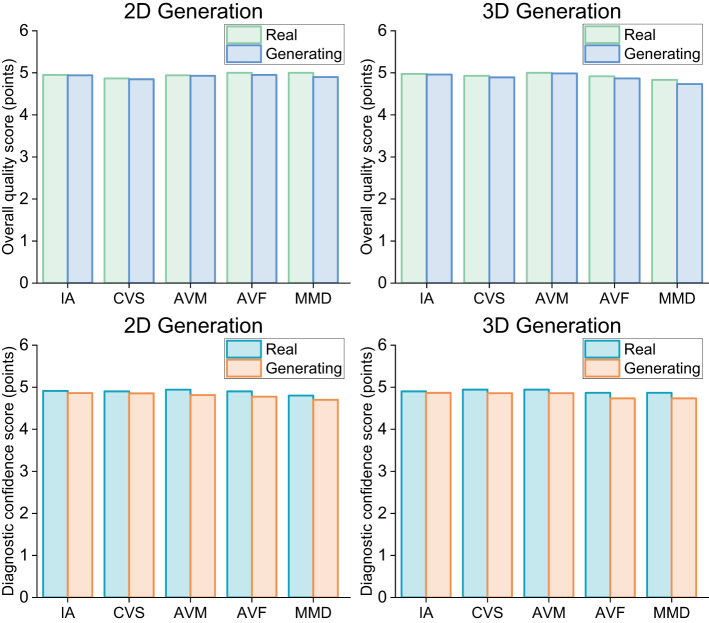
Rating results of 5 interventional radiologists by 5-level scores. **(A)** Assessment results of overall image quality for 2D- and 3D-DSA sequences. **(B)** Assessment results of diagnostic confidence for 2D- and 3D-DSA sequences.

SAVE-Net was evaluated by comparing its generated sequences to real sequences in terms of overall image quality and diagnostic confidence. The detailed evaluation criteria were provided in the Section 3. The summarized scores provided in Supplementary Data 2, Data 3 are illustrated in Figure 5.

The results of the overall image quality (listed in Supplementary Table S6) indicated that the overall scores for the generated sequences (4.919 ± 0.276) were slightly lower than those for the real sequences (4.940 ± 0.237), and this slight difference was observed in both 2D and 3D sequences. In specific categories, the scores for the generated sequences of 2D IA (4.941 ± 0.235), 2D AVM (4.929 ± 0.256), and 3D AVM (4.986 ± 0.119) were very close to those of the real sequences. The scores for the generated sequences in 3D MMD (4.733 ± 0.512) and 3D AVF (4.867 ± 0.340) showed a relatively slight decline but remained above 4.6 points, indicating that although the quality of the model's generated results for these two disease types has decreased, they remain within the clinically acceptable range. Inter-observer agreement in the ratings was assessed using the Kappa coefficient (Supplementary Table S7), and all results were statistically significant (all *P* < 0.001). Among these, the inter-observer agreement for the generated images of 2D AVF, 3D AVF, and 3D MMD was relatively low (0.454, 0.596, and 0.551), indicating that there were some discrepancies in the observers' judgments when evaluating the generated images for these specific disease types. However, the results still demonstrated moderate consistency, suggesting that although the generated results had some shortcomings, they remained effective. Supplementary Table S8 presents the results of paired tests between the real and generated sequences conducted by five observers. The results demonstrated that all *P*-values were greater than 0.05 across all observers in all categories. Therefore, there was no significant difference in overall image quality between the generated and real sequences. This indicates that the model's generated results are comparable to the real sequences in the image quality assessment.

The results of the diagnostic confidence (listed in Supplementary Table S9) represented that the total results of both real and generated sequences achieved high diagnostic confidence scores (4.910 ± 0.293 and 4.838 ± 0.397). In both 2D and 3D modes, the overall scores for the generated sequences (4.836 ± 0.411 and 4.840 ± 0.383) closely approximated those of the real sequences. Within the 2D mode, the diagnostic confidence scores for generated sequences of IA, CVS, and AVF were close to those of the real sequences, with differences of less than 0.1 points (0.053, 0.047, and 0.095). For AVM and MMD, although the differences between generated and real sequences were higher than 0.1 points (0.129 and 0.100), it remained insignificant. Among the various disease types in 3D mode, the score differences for IA, CVS, and AVM were all less than 0.1 points (0.035, 0.086, and 0.086). Meanwhile, the differences for AVF and MMD were both 0.134, indicating that the degree of divergence was comparably small. The results of the inter-observer consistency analysis are shown in Supplementary Table S10. All Kappa coefficients were statistically significant (all *P* < 0.001). For the generated sequences, inter-observer agreement showed an excellent performance, ranging from 0.645 to 1.000. The Kappa values of generated sequences for 2D IA, 2D MMD, 3D IA, and 3D AVF were slightly higher or comparable to those of real sequences. Even in the 3D CVS and 2D AVF categories, where scores were slightly lower, inter-observer agreement for the generated sequences remained within an acceptable range. The results of the Wilcoxon tests indicated that all categories had no significant difference. Therefore, although there exists a slight decrease in the scores for some disease types, this difference remains within an acceptable range.

### 3.6 Evaluation of multi-scenario utilization

**Figure 6:**
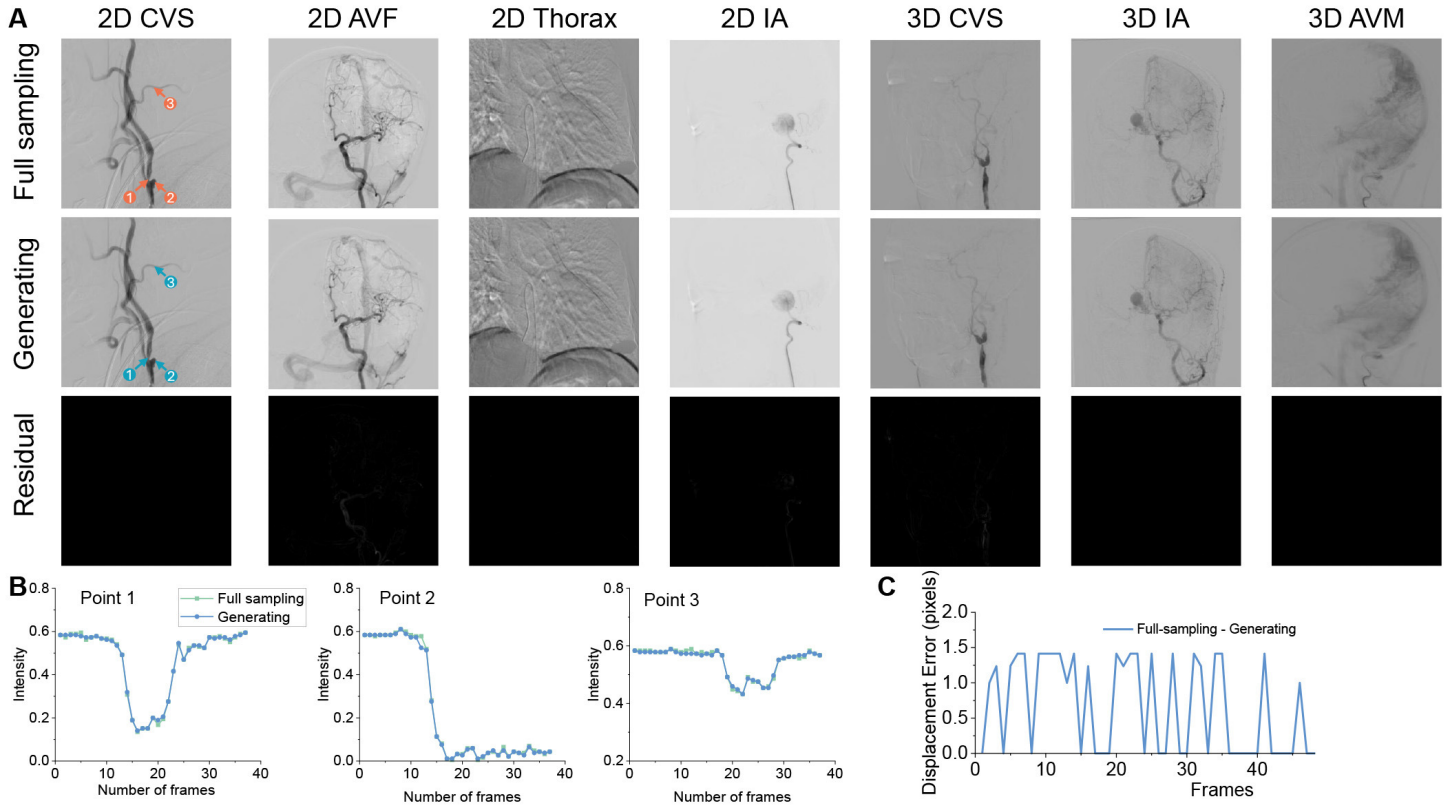
Generative capabilities of SAVE-Net across multiple scenarios. **(A)** Generated results of SAVE-Net in various scenarios. **(B)** The time-intensity curves for the real and generated sequences corresponding to vessel pixels illustrated in **(A)**. **(C)** The displacement curve of the guidewire tip in the generated sequence compared to the real sequence.

The capability of SAVE-Net under different scanning modes, anatomical regions, and disease conditions is illustrated in Figure 6. The generated images accurately reconstruct the dynamic filling process of intracranial vessels under various abnormal states. As indicated by the near absence of signal in the residual maps in Figure 6A, the generation performance is excellent and stable across multiple scenarios. We annotated three random pixels within the stenotic segment, proximal vessel, and distal vessel and plotted the time-intensity curves for both real and generated sequences. The curves demonstrate high consistency between the generated and real sequences (Figure 6B). After normalization to the range [0, 1], the signal intensity error of the generated sequences was below 0.02, meeting the requirements for clinical diagnosis. Furthermore, we measured the displacement error of the guidewire tip in the 2D thorax image shown in Figure 6A, with results presented in Figure 6C. The tip displacement error remained within 1.5 pixels, satisfying the high-precision demands of interventional procedures. Notably, the model maintained robust and clinically applicable reconstruction performance for small AV-shunts. The model achieved an SSIM of 0.974, 0.975, 0.947, and 0.948 for the 2D-AVF, 2D-AVM, 3D-AVF, and 3D-AVM subgroups, respectively, with PSNR consistently above 40 dB for all subgroups. The generated results are shown in Supplementary Figures S2A–C, the results across the whole phases achieved good performance, and the residual signals were not obvious. Time-intensity curve analysis showed that the normalized signal intensity error of the generated sequences was below 0.02, accurately reproducing the rapid contrast filling and washout dynamics of the shunts, which are shown in Supplementary Figures S2D–F. In the visual Turing test, five interventional radiologists were unable to reliably distinguish between the generated and real sequences of these AV-shunt cases (Fleiss' Kappa = 0.089, *P* < 0.05), with no statistically significant difference in overall image quality and diagnostic confidence scores between generated and real sequences (all *P*>0.05).

To further evaluate the performance of SAVE-Net under different artifact conditions, four sequences were qualitatively analyzed, corresponding to Supplementary Figures S3–S5. Supplementary Figure S3 presents a case in 2D-DSA mode where the input frames exhibited severe artifacts at the beginning and end frames, whereas the intermediate frames showed less artifact interference. In the full sampling sequence, vascular structures were not visible in the first and last frames; they appeared transiently as artifacts diminished and disappeared again as artifacts reappeared. In the generated sequence, only faint vascular signals were observed, which were substantially weaker than those in the full sampling sequence, and the artifacts were not correctly eliminated. This observation can be attributed to the absence of discernible vascular structures in the input frames and the lack of significant contrast in artifact components, which represents the ability to effectively identify vascular features. Furthermore, the residual maps exhibited signal residues only in regions corresponding to artifacts, with no detectable vascular residue. This finding indicates that vascular signals were indeed present in the generated results but were obscured by the generated artifacts, while the artifacts themselves were not adequately removed.

Supplementary Figure S4 illustrates another 2D-DSA scenario in which the first frame showed no clear vascular visualization, whereas the last frame displayed clear vascular structures. The vascular structures gradually appeared and became clearer over the course of the sequence. The generated sequence similarly captured this progressive vascular emergence, with no substantial differences in vascular morphology. In the full sampling sequence, the guidewire was clearly visible in the first frame but overlapped with the vessel wall in the last frame, resulting in reduced clarity. In the generated sequence, the annular structure of the guidewire exhibited abnormalities in the early frames due to interference from white artifacts, but it gradually recovered to a normal morphology and position in subsequent frames. The residual maps revealed relatively prominent signal residues near the guidewire, while other regions showed minimal residuals. These results represent that when the generated results contain significant interference and overlapping structures, the model retains a certain capacity for recovery and correction, albeit with some residual differences.

Supplementary Figure S5 presents a 3D-DSA case in which vascular structures were initially unclear due to low contrast between vessel and background, particularly for small vessels. In the generated sequence, the first three frames showed clear vascular structures with well-preserved small vessels. Frames 4 to 6 exhibited slight blurring of small vessels, while frames 7 and 8 showed no significant differences from the full sampling sequence. The residual maps indicated that the main residual signals were concentrated in the dental region, with no substantial residues in vascular areas. These findings represent that SAVE-Net maintained stable performance in generating vascular structures and exerted a certain suppressive effect on non-vascular regions, albeit at the cost of mild image blurring in some frames.

Overall, the results of SAVE-Net match well with the real sequences across various conditions, demonstrating satisfactory accuracy in reproducing vascular morphology, signal intensity, and guidewire motion trajectories. The results from these four DSA sequences demonstrate that SAVE-Net possesses a certain ability to suppress artifacts, which are unwanted distortions in images that can obscure important details. However, when all input frames in a sequence contain severe artifacts, the model cannot effectively eliminate artifacts or accurately generate vascular structures. Furthermore, the artifact-suppression effect may introduce mild image blurring in some frames, but this effect does not cause significant distortion of vascular structures. For sequences with substantial differences between the first and last frames (e.g., gradual vascular filling), SAVE-Net is capable of generating relatively complete DSA images consistent with the normal hemodynamic process, without introducing severe distortions.

### 3.7 Evaluation of generalization performance for out-of distribution sequences

To further assess the out-of-distribution generalization capability of SAVE-Net, we conducted a disease-specific validation. In this experiment, IA, CVS, and AVM data were systematically excluded from both the training and fine-tuning datasets. The model was subsequently trained and fine-tuned using only the remaining disease categories, after which its performance was evaluated on the independent cases of the three excluded diseases drawn from Dataset 2. The quantitative results are summarized in Table 2.

**Table 2:** Quantitative results of cross-category generation.

Categories	SSIM	PSNR
2D IA	0.971 (0.966, 0.980)	42.153 (39.510, 43.602)
2D CVS	0.973 (0.970, 0.980)	42.124 (39.977, 44.842)
2D AVM	0.966 (0.950, 0.970)	41.576 (40.117, 42.841)
3D IA	0.940 (0.929, 0.945)	39.740 (38.009, 41.555)
3D CVS	0.944 (0.934, 0.955)	39.621 (38.289, 41.008)
3D AVM	0.940 (0.930, 0.954)	39.766 (38.339, 40.928)

The findings demonstrate that the absence of these three disease categories during training and fine-tuning did not result in any substantial degradation in model performance. The generated sequences maintained consistent imaging quality and clinical utility. Specifically, in the 2D-DSA mode, the SSIM exhibited only a marginal decline ranging from 0.001 to 0.004, while the PSNR decreased by merely 0.037 to 0.254 dB. These negligible variations confirm that the outcomes for all three disease types remained highly concordant with those obtained from models trained on the complete dataset. In the 3D-DSA mode, the maximum reduction in SSIM was constrained to 0.002, whereas the PSNR exhibited a decrement ranging from 0.737 to 0.892 dB. Although the observed image distortion was marginally higher than that noted in the 2D-DSA mode, it remained confined within a clinically acceptable and narrow range. These results indicate an exceptionally high degree of consistency in the preservation of vascular structural integrity.

Collectively, these findings indicate that the feature extraction capability of SAVE-Net does not depend on memorization of training data tied to specific disease categories. The model, on the other hand, accurately captures the basic idea behind cerebrovascular anatomy. As a result, it is capable of generating DSA sequences that maintain anatomical accuracy and diagnostic validity without requiring dedicated training or fine-tuning for specific or rare pathological conditions. This evidence underscores the robustness and clinical utility of the proposed SAVE-Net.

## 4 Discussion

In this study, we developed and validated SAVE-Net, a deep learning model specifically designed for frame synthesis in digital subtraction angiography (DSA). SAVE-Net integrates three principal modules to address the complex geometric morphology of vascular structures and the spatiotemporal discontinuity problems. First, a Direction-Aware Convolution Block (DACB) constrains convolutional kernels using orientation-specific masks to enhance the network's sensitivity and feature extraction capability for complex vascular structures. Second, a Flow-Guided Frame Synthesis (FGFS) module, built upon bidirectional optical flow estimation, effectively models both the dynamic filling process of the contrast agent and the spatial deformation. This ensures motion continuity and spatial consistency across the generated sequences. Third, a Spatio-Temporal Fusion Bottleneck (STFB) employs inter-frame global attention mechanisms to establish long-term dependencies across both temporal and spatial dimensions, resolving the issue of abrupt changes between frames commonly found in existing generative models. This architecture enables SAVE-Net to generate high-fidelity DSA sequences under low-dose conditions.

Conventional dose-reduction strategies for DSA, such as adjusting tube voltage, current-time product, or reducing field of view (FOV), are constrained by the trade-off between radiation dose and image quality. Previous studies have pointed out that narrowing FOV can improve spatial resolution but will increase the incident air kerma rate on the patient's surface, while adjusting tube parameters often fails to achieve substantial dose reduction without compromising diagnostic utility. In contrast, the experimental results of this study showed that SAVE-Net broke through this limitation. The internal validation utilized 17,335 DSA sequences from Dataset 1 to verify the model's generation capabilities for 1–8 frames and demonstrated that it maintains stable performance across different scanning modes, anatomical structures, and disease types. Furthermore, five physicians with extensive clinical experience conducted a visual Turing test on the generated results ranging from 1 to 8 frames. The results demonstrated that the physicians were unable to effectively distinguish between the generated sequences (1–6 frames) and the real sequences. The experimental results indicate that SAVE-Net can generate up to 6 frames, meaning that a complete and clinically valid DSA sequence can be produced using only 1/7 of the standard radiation dose.

Previous studies such as GenDSA and GaraMoSt have explored the application of generative models in DSA frame synthesis, but the ability of dose reduction is limited, and the generation speed is slow. This study used 3,255 DSA sequences from Dataset 2 obtained from two hospitals to validate the capability of SAVE-Net. The results showed that SAVE-Net achieved a higher average SSIM (0.951 vs. 0.915 of GenDSA and 0.927 of GaraMoSt) and PSNR (40.764 dB vs. 38.313 dB of GenDSA and 38.705 dB of GaraMoSt). Furthermore, the average generation time for SAVE-Net was only 0.212 s, which is approximately 2.1 times faster than that of the other two models. Visual comparison results also showed that the image generated by SAVE-Net had clearer vascular structures, while GenDSA and GaraMoSt had obvious residual signals. These experiment results fully confirmed that SAVE-Net had more outstanding performance than existing models in balancing image quality, generation efficiency, and clinical applicability.

To further validate the clinical utility of the model, this study conducted a clinical assessment of the generated results based on overall image quality and diagnostic confidence. In the evaluation of overall image quality, the scores of the generated sequences were highly similar to those of the real sequences, and the Kappa coefficients indicated that the model demonstrated high consistency across all categories except for 2D AVF, while the Kappa coefficient for the 2D AVF disease type reached 0.454, indicating a modest consistency of inter-observer agreement. Furthermore, the *P*-values for inter-observer comparisons across categories were less than 0.001, demonstrating that the scores had no significant difference. The *P*-values for each observer's scoring of the generated and real sequences were all greater than 0.05, indicating the validity of the model's generated results. For the results of the diagnostic confidence, the results of the generated and real sequences also had no significant difference, and the scores were highly similar. Although the scores differences between the generated and real sequences for 2D AVM, 2D MMD, 3D AVF, and 3D MMD were no less than 0.1 points, the scores remained significantly higher than 4.6 points, indicating the validity and clinical utility of the generated sequences. Moreover, the inter-observer Kappa coefficients were all greater than 0.5, with P-values all less than 0.001, indicating a high consistency of inter-observer agreement. The results of the Wilcoxon test showed no significant difference between the generated and real sequences. These findings demonstrate that the model's generated results possess high clinical utility for diagnostic purposes.

This study further utilized a comprehensive multi-scenario validation to assess the model's robustness under various clinical conditions and challenging cases. Across different scanning modes (2D- and 3D-DSA) and anatomical regions, SAVE-Net produced sequences that were highly consistent with real sequences. The authenticity of fine details was quantitatively corroborated by analysis of time-intensity curves and measurement of guidewire tip displacement error. For arteriovenous shunts (AV-shunts), which exhibit extremely short imaging durations, the sequences generated by SAVE-Net not only deliver excellent overall quantitative results, but the time-intensity curves also show that the signal intensity error of the generated sequences is less than 0.02 throughout the entire phases from the arterial phase to the venous phase. This accurately reproduces key transient hemodynamic characteristics, demonstrating that the generated sequences maintain effective fidelity in terms of spatial intensity and temporal consistency. The impact of image artifacts on model performance was systematically analyzed across varying degrees of artifact severity and scenarios with large discrepancies between input frames. The analysis revealed that the model's performance remains robust provided that at least one input frame contains a sufficiently clear depiction of the vascular anatomy. Only in extreme cases where severe artifacts entirely obscure vascular structures across all input frames does the model fail to reconstruct a clear vascular depiction. Conversely, when substantial differences exist between input frames (e.g., due to progressive contrast filling), SAVE-Net reliably generates a complete sequence that accurately reflects the normal hemodynamic process, exhibiting no discernible deviation from the real sequence. These findings delineate the operational boundaries of the model, confirming that it can generate valid and realistic sequences as long as the input frames are free from severe, vessel-obscuring artifacts. Finally, the model's out-of-distribution generalization capability was rigorously evaluated by excluding three disease categories characterized by significant cerebrovascular abnormalities, IA, CVS, and AVM, from both the training and fine-tuning datasets. Quantitative results from this cross-category validation demonstrated that, for all three excluded disease types across both 2D and 3D scanning modes, SAVE-Net was able to generate valid and realistic sequences without any prior exposure to these specific pathologies during model development. This finding holds significant clinical implications, suggesting that SAVE-Net does not rely on memorization of disease-specific patterns. Instead, it learns generalizable representations of cerebrovascular anatomy and hemodynamics. Consequently, the model is poised to generate diagnostically useful sequences even when encountering novel disease presentations or rare anatomical variants in clinical practice, thereby eliminating the need for retraining or fine-tuning on such emergent cases.

This study still has some limitations that need to be addressed in future research. First, although the experimental results demonstrate that the sequences generated by SAVE-Net closely resemble real sequences, a downward trend and lower Kappa coefficient exist for some disease types in the clinical assessments. Future studies should incorporate data with more complex vascular structures to further investigate whether the current problems are random and to conduct a more sufficient evaluation and implement relevant improvements. Furthermore, this study demonstrated that SAVE-Net achieved an excellent performance in some anatomical regions like head, neck, and thorax, but the number of anatomical structures analyzed was limited. Therefore, further research should include more regions such as the heart and limbs to further expand the clinical applicability of SAVE-Net. In addition, although experiments were conducted across various clinical scenarios to demonstrate the model's generalizability and robustness, it has not yet been evaluated on real-world clinical tasks. In the future, we could incorporate tasks such as IA segmentation and CVS detection using generated sequences to further indicate the model's applicability to downstream tasks.

## 5 Conclusion

This study proposes SAVE-Net, a deep learning-based low-dose DSA imaging model that generates high-fidelity DSA sequences using only 1/7 of the standard radiation dose through a novel frame synthesis technique. The research demonstrates the viability of an ultra-low-dose imaging framework that directly responds to the clinical demand of reducing radiation dose while preserving diagnostic utility and image quality. The experimental results indicated that SAVE-Net can be used in a wide range of imaging systems, anatomical areas, and cerebrovascular diseases, while keeping the reality of the generation results with only 1/7 of the usual radiation dose. The framework not only achieves state-of-the-art performance in image similarity metrics, but it also guarantees temporal coherence and anatomical precision needed for interventional guidance, as shown by strict evaluations by multiple readers. This method provides a practical and hardware-independent means to enhance the safety of patients and operators in interventional neuroradiology.

## Data Availability

The data analyzed in this study is subject to the following licenses/restrictions: The data that support the findings of this study are available on reasonable request from the corresponding author. Requests to access these datasets should be directed to He Ma, mahe@bmie.neu.edu.cn.
