# Supplementary material for: Deep learning-based frame synthesis enables radiation dose reduction in digital subtraction angiography imaging: a multicenter study
Source: Front Med (Lausanne). 2026 May 15;13:1793962. doi: 10.3389/fmed.2026.1793962 (PMC13219322; doi:10.3389/fmed.2026.1793962)
Supplement: Supplementary file 1 [file Supplementary_File_1.PDF]

Supplementary Material

1 MODEL DETAILS

1.1 Multi-scale feature encoder

The MFE extracted multi-scale feature representations from two input DSA frames $I_1, I_2 \in \mathbb{R}^{B\times 1\times H\times W}$, where B denoted the batch size, and H and W represented the height and width of the input image. We concatenated the input frames along the batch dimension to prevent premature feature fusion before the frame estimation process. The encoder consisted of six stages, which implemented a progressive downsampling strategy through convolutional layers with a stride of 2. This design captured a rich hierarchy of features, from local details to global semantics. To prevent unintended feature interactions between the two input frames prior to the synthesis of the new frame, we maintained independent feature extraction paths for each frame. Each stage contained multiple Direction-Aware Convolution Blocks (DACB), which enhanced the model's sensitivity to directional vascular features. The subsequent subsections detail the direction-aware convolution operator and the DACB structure.

1.1.1 Direction-aware convolutional kernel

Since standard convolutional structures struggle to model the complex geometry of vascular networks in DSA images, we introduced a direction-aware convolution to incorporate explicit directional priors. We defined a directional mask $M \in \{0,1\}^{K_h\times K_w}$ to constrain the convolution kernel $W_c \in \mathbb{R}^{C_{out}\times C_{in}\times K_h\times K_w}$ along specific orientations via element-wise multiplication:

$$W_{dir} = M \odot W_c \tag{S1}$$

We constructed convolutional kernels for four primary directions that cover the main distribution orientations of cerebrovascular structures in DSA images, with the corresponding non-trainable directional masks.

The horizontal direction mask constrains kernel weights to the horizontal row, enhancing feature extraction for horizontally distributed vessels.

$$M_{horizontal} = \begin{bmatrix} 0 & 0 & 0 \\ 1 & 1 & 1 \\ 0 & 0 & 0 \end{bmatrix} \tag{S2}$$

The vertical direction mask constrains kernel weights to the vertical column, enhancing feature extraction for vertically distributed vessels.

$$M_{vertical} = \begin{bmatrix} 0 & 1 & 0 \\ 0 & 1 & 0 \\ 0 & 1 & 0 \end{bmatrix} \tag{S3}$$

The principal diagnal direction mask constrains kernel weights to the main diagnal, enhancing feature extraction for vessels along this orientation.

$$M_{pri-diag} = \begin{bmatrix} 1 & 0 & 0 \\ 0 & 1 & 0 \\ 0 & 0 & 1 \end{bmatrix} \tag{S4}$$

The anti-diagnal direction mask constrains kernel weights to the anti-diagnal, enhancing feature extraction for vessels along this orientation.

$$M_{pri-diag} = \begin{bmatrix} 0 & 0 & 1 \\ 0 & 1 & 0 \\ 1 & 0 & 0 \end{bmatrix} \tag{S5}$$

The directional mask is multiplied element-wise with the original learnable convolution kernel to obtain the direction-aware convolution kernel. The kernel weights are updated synchronously during model training, while the directional mask remains fixed to retain the explicit directional prior.

1.1.2 Direction-aware convolution block

The DACB is specifically optimized for the imaging characteristics of DSA, targeting two core pain points of standard convolution in vascular feature extraction: (1) Standard convolutional kernels lack explicit directional prior constraints, resulting in insufficient sensitivity to the strip-shaped, multi-directionally distributed tiny vascular structures in DSA images, especially the transiently visible shunt structures in AV-Shunt lesions; (2) Continuous C-arm rotation in 3D-DSA leads to dynamic changes in vascular projection direction, which requires the convolution module to maintain stable feature extraction performance for vessels under different viewing angles.

The core of DACB is the direction-aware convolutional kernel, which introduces explicit directional priors by constraining the weight distribution of the convolution kernel along specific vascular orientations via a directional mask. The block adopts a 4-branch parallel design, with each branch focusing on feature extraction along one primary vascular direction, and integrates a channel attention mechanism to adaptively enhance vascular-related discriminative features. It first applied depth-wise convolution to the input features f_{in} and then split the output into N groups ($N = 4$) along the channel dimension. Each group passed through a directional convolution branch that incorporated channel attention. Finally, the block concatenated the outputs from all branches and fused them using a point-wise convolution. The computation proceeded as follows:

$$\{f_i\}_{i=1}^{N} = \text{Split}(\text{DWConv}(f_{in})) \tag{S6}$$

$$\alpha_i = \sigma(W_2 \cdot \phi(W_{dir}^{i}\phi(W_1 \cdot f_i))) \tag{S7}$$

$$f_i' = \alpha i \odot f_i \tag{S8}$$

$$f_{out} = W_3 \cdot \phi(\text{Concat}(\{f_i\}_{i=1}^{N})) + W_4 \cdot f_{in} \tag{S9}$$

Here, DWConv and Split denoted depth-wise convolution and channel-wise split operations, respectively; W_1, W_2, W_3, W_4 were 1×1 convolutional layers; W_{dir}^{i} was the directional convolutional

layer for the i-th branch; α_i represented the attention weight; and σ and ϕ were the Sigmoid and GELU activation functions, respectively.

This design enables the model to maintain high sensitivity to tiny vascular structures under different projection angles, and effectively adapts to the dynamic changes of vascular morphology in both 2D-DSA and 3D-DSA sequences, laying a core foundation for the high-fidelity frame synthesis of SAVE-Net.

1.2 Flow-guided frame synthesis module

The FGFS module addressed challenges such as brightness variation and vascular occlusion by estimating bidirectional optical flow and refining the synthesized features. This module consisted of four components: a Bidirectional Optical Flow Network (BOFNet), a differentiable image warping operation, a Feature Refinement Network (FRN), and a Direction-Aware Transformer (DaT). We also incorporated temporal step conditioning to ensure consistency across generated frames.

1.2.1 Bidirectional optical flow network

We designed BOFNet to predict both forward ($F_{1\to2}$) and backward ($F_{2\to1}$) optical flow fields, each of size $\mathbb{R}^{B\times2\times H\times W}$. The network comprised an input projection layer using point-wise convolution, a feature embedding module with two residual blocks, and two independent pixel-wise convolution heads for flow prediction. This bidirectional design provided richer motion information and improved the handling of occluded regions.

1.2.2 Differentiable image warping

We utilized the estimated bidirectional flow fields to warp the input frame features via bilinear interpolation, which ensured gradient propagation during training. We then fused the warped features from both directions through averaging to produce the initial synthesized frame.

1.2.3 Feature refinement network

The FRN corrected artifacts caused by optical flow inaccuracies and occlusions. It took the original frame features, the warped features, and the bidirectional optical flow fields as input:

$$f_{out} = \mathcal{G}(\text{Concat}(f_1, f_2, f_1^w, f_2^w, f_{1\to2}, f_{2\to1}) \tag{S10}$$

where $\mathcal{G}$ was implemented as a Convolutional Multilayer Perceptron (ConvMLP). We incorporated the original frame features via residual connections to mitigate information loss and used a timestep embedding to maintain temporal coherence.

1.2.4 Direction-aware transformer

To further enhance the quality of the synthesized frames, we employed the DaT module, which combined directional convolution with global self-attention. We applied DaT only to deep features due to the prohibitive computational cost of processing large shallow feature maps and the greater relevance of structural abstraction at deeper layers. DaT first extracted features along four distinct orientations using directional convolutions, concatenated the results, and applied a channel shuffle operation. A standard transformer encoder then modeled long-range dependencies to generate the refined output.

1.3 Spatio-temporal fusion bottleneck

The STFB integrated multi-frame information through a cascade of three components. We first used two DaT modules to extract global spatial features from the two input frames. We then applied the FGFS module to generate the features of the new intermediate frame. Finally, we incorporated two Frame Transformer (FT) modules to capture global inter-frame dependencies and model interactions across the entire frame sequence.

1.3.1 Frame transformer

The FT module modeled joint spatio-temporal relationships across multiple frames. We embedded spatial features into sequences using a convolutional patch embedding module. The module then projected these sequences into queries, keys, and values via linear transformations, while applying Rotary Position Embedding (RoPE) (Su et al., 2024) to incorporate positional information. Finally, it computed attention following the standard transformer architecture. We merged the frame dimension into the channel dimension, enabling efficient simultaneous spatial and inter-frame attention without introducing additional computational complexity.

1.4 Multi-scale feature decoder

The MFD reconstructed the output frames through progressive upsampling. It performed multi-scale fusion between the output features of the bottleneck layer f_b and the output features of the FGFS module f_d using skip connections. Each upsampling stage used nearest-neighbor interpolation followed by a convolutional layer, and we integrated f_b and f_d using two DACB modules. The final output features were generated through a point-wise convolution, and we constrained the pixel values of the prediction to the range $[-1, 1]$ using the Tanh activation function.

2 LOSS FUNCTIONS

We defined the total training loss as a weighted combination of four components:

$$\mathcal{L}_{total} = \alpha \cdot \mathcal{L}_{pixel} + \beta \cdot \mathcal{L}_{struct} + \gamma \cdot \mathcal{L}_{feat} + \delta \cdot \mathcal{L}_{style} \tag{S11}$$

where α, β, γ, and δ were weighting coefficients. The pixel reconstruction loss $\mathcal{L}_{pixel}$ was the L1-norm between the generated and ground truth DSA sequences. The structural preservation loss $\mathcal{L}_{struct}$ was $1 - \text{SSIM}(\text{pred}, \text{gt})$, where pred and gt are the prediction and ground truth.

The feature matching loss $\mathcal{L}_{feat}$ and style consistency loss $\mathcal{L}_{style}$ were calculated on multi-scale features $\{f_i\}_{i=1}^{4}$. ResNet34 was used as the feature extractor, which pretrained on ImageNet, without any adaptation or fine-tuning. These loss functions are defined as:

$$\mathcal{L}_{feat} = \sum_{n=1}^{N} \sum_{l=1}^{4} \frac{1}{N_l} \| f_{l,n}^{pred} - f_{l,n}^{gt} \|_1 \tag{S12}$$

$$\mathcal{L}_{style} = \sum_{n=1}^{N} \sum_{l=1}^{4} \frac{1}{N_l} \| f_{l,n}^{pred} - f_{l,n}^{gt} \|_2^2 \tag{S13}$$

Here, N was the number of frames, N_l was the number of pixels in the l-th feature map, and $f_{l,n}^{pred}$ and $f_{l,n}^{gt}$ were the predicted and ground-truth features at the l-th layer for the n-th frame,

respectively. ResNet34 was pretrained on the ImageNet dataset as a fixed feature extractor without any adoption or fine-tuning process.

We employed a two-phase training strategy for the loss weights. During the initial 50 epochs, we set $(\alpha, \beta, \gamma, \delta) = (1, 1, 0, 0)$ to establish a strong baseline via pixel-level and structural constraints. For the remaining epochs, we adjusted the weights to $(0.5, 0.7, 40, 40)$ to enhance semantic and stylistic consistency.

3 MANUAL ASSESSMENT CRITERIA

Evaluations were conducted on both the generated results and the real sequences with respect to two key aspects: overall image quality and diagnostic confidence. The corresponding scoring criteria are detailed in Table S1 and Table S2. The scoring system of diagnostic confidence is formulated based on the clinical diagnostic workflow of neurointerventional radiology, and the scoring is completely oriented to whether the sequence can support reliable clinical diagnosis and decision-making, rather than simple image quality evaluation.

Table S1. Overall image quality scoring criteria

Score	Criteria
1	1. Artifacts, noise or distortion rendering the image completely unreadable;<br>2. Severely imbalanced contrast/brightness, making structure differentiation impossible;<br>3. Complete absence of key anatomical regions;<br>4. Insufficient video smoothness with obvious abrupt changes.
2	1. Severe artifacts, noise or distortion that interfere with observation;<br>2. Poor contrast/brightness, with key structures displayed blurrily;<br>3. Missing important anatomical structures or incorrect localization;<br>4. Insufficient video smoothness or obvious abrupt changes.
3	1. Obvious but not severe artifacts, noise or distortion;<br>2. Slightly poor contrast/brightness, but key structures still identifiable;<br>3. Possible minor imperfections in localization;<br>4. Slightly unsmooth video with minor abrupt changes.
4	1. Slight artifacts or noise present, but not affecting diagnosis;<br>2. Moderate contrast and brightness, with key structures displayed clearly;<br>3. Complete coverage of anatomical structures;<br>4. Smooth video with minor abrupt changes.
5	1. No artifacts or noise interference of any kind;<br>2. Optimal contrast and brightness, with all key structures displayed extremely clearly;<br>3. Complete anatomical structure localization without omission;<br>4. Smooth video with no abrupt changes.

Table S2. Diagostic confidence scoring criteria

Score	Criteria
1	1. Unable to identify normal or abnormal structures;<br>2. Cannot provide any meaningful diagnostic opinions.
2	1. Keyframes or key features are blurred or missing;<br>2. Connot make a reliable diagnostic judgement.
3	1. There exist interfering factors that affect the judgement;<br>2. Multiple possibilities exist or the diagnosis remains questionable.
4	1. Keyframes or key features are clear;<br>2. A relatively confident diagnosis can be reached, while other potentialities still exist.
5	1. Keyframes and key features are clear and unambiguous;<br>2. There is no room for doubt about the diagnosis, and no alternative possibilities exist.

4 SUPPLEMENTARY TABLES AND FIGURES

4.1 Tables

Table S3: Quantitative results of SAVE-Net for different number of frames.

Frames	Categories	SSIM	PSNR (dB)	Time (s)
1	2D IA	0.981 (0.977, 0.985)	43.879 (43.365, 44.392)	0.125 (0.099, 0.151)
	2D CVS	0.983 (0.981, 0.986)	44.484 (43.777, 45.191)	0.113 (0.088, 0.138)
	2D AVM	0.981 (0.978, 0.984)	44.236 (43.723, 44.749)	0.142 (0.116, 0.167)
	2D AVF	0.983 (0.981, 0.985)	44.237 (43.813, 44.661)	0.126 (0.103, 0.150)
	2D MMD	0.980 (0.974, 0.986)	44.097 (43.525, 44.669)	0.110 (0.090, 0.131)
	2D Thorax	0.982 (0.978, 0.985)	43.918 (43.545, 44.290)	0.133 (0.108, 0.159)
	2D Total	0.982 (0.980, 0.983)	44.139 (43.945, 44.332)	0.125 (0.115, 0.134)
	3D IA	0.958 (0.954, 0.961)	42.165 (41.802, 42.528)	0.115 (0.090, 0.141)
	3D CVS	0.956 (0.952, 0.959)	41.987 (41.619, 42.354)	0.122 (0.099, 0.145)
	3D AVM	0.958 (0.954, 0.962)	41.969 (41.582, 42.356)	0.104 (0.082, 0.126)
	3D AVF	0.957 (0.954, 0.961)	42.042 (41.669, 42.416)	0.140 (0.115, 0.165)
	3D MMD	0.958 (0.955, 0.961)	41.948 (41.523, 42.374)	0.118 (0.097, 0.139)
	3D Total	0.957 (0.956, 0.959)	42.022 (41.856, 42.188)	0.120 (0.110, 0.130)
	Total	0.964 (0.962, 0.965)	42.568 (42.397, 42.739)	0.123 (0.116, 0.129)
2	2D IA	0.979 (0.975, 0.982)	43.309 (42.973, 43.646)	0.145 (0.114, 0.175)
	2D CVS	0.980 (0.977, 0.984)	43.280 (42.865, 43.694)	0.132 (0.103, 0.161)
	2D AVM	0.979 (0.976, 0.982)	43.018 (42.538, 43.498)	0.166 (0.135, 0.196)
	2D AVF	0.982 (0.981, 0.984)	43.617 (43.252, 43.983)	0.147 (0.120, 0.175)
	2D MMD	0.979 (0.975, 0.983)	43.266 (43.015, 43.516)	0.129 (0.104, 0.153)
	2D Thorax	0.981 (0.978, 0.983)	43.485 (43.184, 43.786)	0.156 (0.126, 0.186)
	2D Total	0.980 (0.979, 0.981)	43.331 (43.191, 43.471)	0.146 (0.134, 0.157)
	3D IA	0.954 (0.950, 0.957)	41.619 (41.242, 41.995)	0.135 (0.105, 0.165)

Continued on next page

Table S3 – Continued from previous page

Frames	Categories	SSIM	PSNR (dB)	Time (s)
	3D CVS	0.957 (0.954, 0.960)	41.801 (41.467, 42.136)	0.143 (0.116, 0.169)
	3D AVM	0.954 (0.951, 0.957)	41.345 (41.043, 41.647)	0.120 (0.095, 0.146)
	3D AVF	0.953 (0.950, 0.956)	41.252 (40.898, 41.607)	0.163 (0.134, 0.192)
	3D MMD	0.954 (0.951, 0.957)	41.432 (41.102, 41.762)	0.136 (0.112, 0.160)
	3D Total	0.954 (0.953, 0.956)	41.490 (41.340, 41.640)	0.139 (0.128, 0.151)
	Total	0.961 (0.959, 0.963)	41.964 (41.821, 42.106)	0.143 (0.135, 0.151)
3	2D IA	0.980 (0.977, 0.982)	42.906 (42.545, 43.267)	0.165 (0.130, 0.200)
	2D CVS	0.977 (0.973, 0.981)	42.570 (42.193, 42.947)	0.150 (0.117, 0.184)
	2D AVM	0.973 (0.959, 0.987)	42.291 (41.596, 42.985)	0.189 (0.155, 0.224)
	2D AVF	0.941 (0.864, 1.018)	41.202 (38.103, 44.302)	0.168 (0.136, 0.199)
	2D MMD	0.980 (0.978, 0.982)	42.986 (42.391, 43.581)	0.146 (0.119, 0.174)
	2D Thorax	0.981 (0.979, 0.984)	43.310 (42.776, 43.844)	0.177 (0.144, 0.211)
	2D Total	0.972 (0.960, 0.984)	42.551 (42.031, 43.071)	0.166 (0.153, 0.179)
	3D IA	0.955 (0.952, 0.958)	41.400 (41.068, 41.733)	0.154 (0.119, 0.188)
	3D CVS	0.952 (0.949, 0.956)	41.259 (40.932, 41.587)	0.163 (0.132, 0.193)
	3D AVM	0.952 (0.949, 0.955)	41.138 (40.889, 41.387)	0.138 (0.108, 0.168)
	3D AVF	0.953 (0.950, 0.956)	41.295 (40.948, 41.641)	0.187 (0.154, 0.221)
	3D MMD	0.952 (0.949, 0.955)	41.041 (40.728, 41.353)	0.157 (0.129, 0.184)
	3D Total	0.953 (0.952, 0.954)	41.227 (41.088, 41.365)	0.160 (0.146, 0.173)
	Total	0.958 (0.954, 0.961)	41.567 (41.391, 41.743)	0.163 (0.154, 0.172)
4	2D IA	0.977 (0.973, 0.981)	42.696 (42.399, 42.993)	0.187 (0.147, 0.226)
	2D CVS	0.980 (0.978, 0.982)	42.686 (42.183, 43.189)	0.170 (0.132, 0.207)
	2D AVM	0.980 (0.977, 0.982)	42.411 (41.853, 42.970)	0.213 (0.174, 0.253)
	2D AVF	0.976 (0.973, 0.980)	42.041 (41.733, 42.349)	0.190 (0.154, 0.226)
	2D MMD	0.979 (0.976, 0.983)	43.022 (42.405, 43.640)	0.165 (0.134, 0.196)
	2D Thorax	0.977 (0.975, 0.979)	42.202 (41.797, 42.607)	0.201 (0.162, 0.239)
	2D Total	0.978 (0.977, 0.979)	42.505 (42.320, 42.689)	0.188 (0.173, 0.202)
	3D IA	0.952 (0.949, 0.954)	40.995 (40.700, 41.289)	0.173 (0.134, 0.212)
	3D CVS	0.951 (0.949, 0.954)	41.017 (40.731, 41.302)	0.184 (0.149, 0.218)
	3D AVM	0.953 (0.950, 0.956)	41.083 (40.761, 41.404)	0.156 (0.122, 0.189)
	3D AVF	0.952 (0.949, 0.955)	40.985 (40.690, 41.279)	0.211 (0.173, 0.249)
	3D MMD	0.951 (0.948, 0.954)	41.055 (40.738, 41.372)	0.176 (0.145, 0.208)
	3D Total	0.952 (0.951, 0.953)	41.027 (40.894, 41.160)	0.180 (0.165, 0.195)
	Total	0.959 (0.957, 0.960)	41.408 (41.283, 41.532)	0.184 (0.174, 0.195)
5	2D IA	0.978 (0.976, 0.981)	41.879 (41.350, 42.408)	0.208 (0.164, 0.253)
	2D CVS	0.979 (0.977, 0.981)	42.207 (41.523, 42.891)	0.190 (0.147, 0.232)
	2D AVM	0.976 (0.972, 0.980)	41.932 (41.456, 42.408)	0.239 (0.195, 0.283)
	2D AVF	0.976 (0.973, 0.978)	42.016 (41.643, 42.389)	0.212 (0.172, 0.252)
	2D MMD	0.974 (0.970, 0.979)	42.104 (41.671, 42.537)	0.185 (0.150, 0.220)
	2D Thorax	0.977 (0.974, 0.979)	42.014 (41.612, 42.416)	0.225 (0.181, 0.268)
	2D Total	0.977 (0.975, 0.978)	42.025 (41.838, 42.212)	0.210 (0.193, 0.226)
	3D IA	0.951 (0.948, 0.953)	40.487 (40.210, 40.764)	0.194 (0.150, 0.238)

Continued on next page

Table S3 – Continued from previous page

Frames	Categories	SSIM	PSNR (dB)	Time (s)
	3D CVS	0.947 (0.944, 0.950)	40.440 (40.142, 40.737)	0.205 (0.167, 0.244)
	3D AVM	0.950 (0.947, 0.953)	40.735 (40.351, 41.119)	0.174 (0.136, 0.212)
	3D AVF	0.951 (0.948, 0.954)	40.682 (40.405, 40.959)	0.236 (0.193, 0.279)
	3D MMD	0.950 (0.947, 0.953)	40.494 (40.196, 40.793)	0.197 (0.162, 0.232)
	3D Total	0.950 (0.948, 0.951)	40.568 (40.431, 40.704)	0.201 (0.184, 0.218)
	Total	0.957 (0.955, 0.958)	40.942 (40.816, 41.068)	0.206 (0.194, 0.218)
6	2D IA	0.979 (0.977, 0.981)	41.854 (41.328, 42.380)	0.229 (0.180, 0.278)
	2D CVS	0.977 (0.973, 0.980)	42.130 (41.755, 42.504)	0.208 (0.161, 0.255)
	2D AVM	0.974 (0.971, 0.976)	41.459 (41.145, 41.772)	0.263 (0.214, 0.311)
	2D AVF	0.976 (0.972, 0.979)	41.814 (41.443, 42.185)	0.233 (0.189, 0.277)
	2D MMD	0.975 (0.972, 0.979)	42.222 (41.683, 42.760)	0.204 (0.165, 0.242)
	2D Thorax	0.975 (0.971, 0.979)	42.004 (41.548, 42.461)	0.248 (0.200, 0.295)
	2D Total	0.976 (0.975, 0.977)	41.914 (41.743, 42.086)	0.231 (0.213, 0.249)
	3D IA	0.948 (0.945, 0.951)	40.256 (39.995, 40.517)	0.214 (0.165, 0.262)
	3D CVS	0.948 (0.945, 0.950)	40.386 (40.139, 40.633)	0.226 (0.184, 0.269)
	3D AVM	0.950 (0.946, 0.953)	40.774 (40.401, 41.147)	0.191 (0.150, 0.233)
	3D AVF	0.948 (0.945, 0.951)	40.411 (40.116, 40.706)	0.260 (0.213, 0.308)
	3D MMD	0.950 (0.948, 0.953)	40.597 (40.271, 40.923)	0.217 (0.178, 0.256)
	3D Total	0.949 (0.947, 0.950)	40.485 (40.350, 40.619)	0.222 (0.203, 0.241)
	Total	0.956 (0.954, 0.957)	40.851 (40.729, 40.973)	0.227 (0.214, 0.240)
7	2D IA	0.975 (0.971, 0.979)	41.660 (41.065, 42.255)	0.251 (0.197, 0.305)
	2D CVS	0.975 (0.971, 0.978)	41.530 (41.060, 42.001)	0.228 (0.177, 0.280)
	2D AVM	0.976 (0.973, 0.978)	41.912 (41.313, 42.511)	0.288 (0.235, 0.342)
	2D AVF	0.974 (0.970, 0.977)	41.572 (41.089, 42.054)	0.256 (0.207, 0.305)
	2D MMD	0.975 (0.972, 0.977)	41.689 (41.355, 42.023)	0.223 (0.180, 0.265)
	2D Thorax	0.973 (0.970, 0.977)	41.001 (40.484, 41.518)	0.271 (0.219, 0.323)
	2D Total	0.974 (0.973, 0.976)	41.556 (41.357, 41.755)	0.253 (0.233, 0.273)
	3D IA	0.947 (0.944, 0.950)	40.181 (39.910, 40.452)	0.234 (0.181, 0.287)
	3D CVS	0.945 (0.942, 0.948)	40.096 (39.786, 40.405)	0.247 (0.200, 0.294)
	3D AVM	0.948 (0.945, 0.951)	40.312 (39.959, 40.666)	0.209 (0.163, 0.255)
	3D AVF	0.949 (0.946, 0.952)	40.444 (40.168, 40.720)	0.285 (0.233, 0.336)
	3D MMD	0.947 (0.944, 0.950)	40.095 (39.734, 40.456)	0.238 (0.195, 0.280)
	3D Total	0.947 (0.946, 0.949)	40.226 (40.086, 40.365)	0.242 (0.222, 0.263)
	Total	0.954 (0.953, 0.956)	40.566 (40.440, 40.693)	0.248 (0.234, 0.262)
8	2D IA	0.973 (0.968, 0.978)	40.996 (40.394, 41.597)	0.272 (0.214, 0.331)
	2D CVS	0.974 (0.971, 0.977)	41.247 (40.859, 41.634)	0.248 (0.192, 0.304)
	2D AVM	0.974 (0.971, 0.978)	41.062 (40.324, 41.801)	0.313 (0.255, 0.371)
	2D AVF	0.971 (0.969, 0.974)	41.199 (40.849, 41.548)	0.278 (0.225, 0.331)
	2D MMD	0.972 (0.969, 0.976)	41.139 (40.671, 41.607)	0.242 (0.196, 0.288)
	2D Thorax	0.975 (0.972, 0.978)	41.347 (40.873, 41.821)	0.295 (0.238, 0.352)
	2D Total	0.973 (0.972, 0.975)	41.167 (40.970, 41.364)	0.275 (0.253, 0.296)
	3D IA	0.949 (0.946, 0.952)	40.327 (39.983, 40.672)	0.254 (0.196, 0.312)

Continued on next page

Table S3 – Continued from previous page

Frames	Categories	SSIM	PSNR (dB)	Time (s)
	3D CVS	0.946 (0.943, 0.948)	39.928 (39.647, 40.208)	0.269 (0.218, 0.320)
	3D AVM	0.948 (0.945, 0.951)	40.168 (39.824, 40.512)	0.227 (0.178, 0.277)
	3D AVF	0.947 (0.944, 0.949)	40.108 (39.797, 40.420)	0.310 (0.253, 0.366)
	3D MMD	0.948 (0.945, 0.950)	40.095 (39.797, 40.394)	0.259 (0.212, 0.305)
	3D Total	0.947 (0.946, 0.949)	40.125 (39.986, 40.265)	0.264 (0.241, 0.286)
	Total	0.954 (0.953, 0.955)	40.391 (40.270, 40.513)	0.270 (0.254, 0.285)

Table S4. Consistency analysis for the results of different frame numbers.

Frames	Kappa	P value
1	0.020	< 0.05
2	0.055	< 0.05
3	0.106	< 0.05
4	0.023	< 0.05
5	0.118	< 0.05
6	0.108	< 0.05
7	0.126	0.06
8	0.101	0.790

Table S5. Comparison results for 6-frame generation sequences.

Models	Categories	SSIM	PSNR (dB)	Time (s)
GaraMoSt	2D IA	0.962 (0.960, 0.965)	40.265 (39.909, 40.622)	0.809 (0.808, 0.809)
	2D CVS	0.957 (0.953, 0.960)	40.232 (39.890, 40.574)	0.702 (0.679, 0.725)
	2D AVM	0.966 (0.965, 0.968)	40.703 (40.350, 41.055)	0.671 (0.671, 0.671)
	2D AVF	0.965 (0.963, 0.966)	40.514 (40.168, 40.861)	0.377 (0.307, 0.447)
	2D MMD	0.969 (0.968, 0.971)	41.223 (40.902, 41.543)	0.274 (0.274, 0.274)
	2D Thorax	0.965 (0.963, 0.967)	40.004 (39.656, 40.352)	0.274 (0.274, 0.274)
	2D Total	0.964 (0.963, 0.965)	40.487 (40.346, 40.628)	0.518 (0.482, 0.553)
	3D IA	0.909 (0.907, 0.911)	37.783 (37.667, 37.899)	0.274 (0.274, 0.274)
	3D CVS	0.912 (0.911, 0.914)	38.005 (37.897, 38.112)	0.295 (0.275, 0.316)
	3D AVM	0.907 (0.905, 0.908)	37.670 (37.564, 37.776)	0.416 (0.416, 0.416)
	3D AVF	0.908 (0.906, 0.910)	37.774 (37.666, 37.881)	0.416 (0.416, 0.416)
	3D MMD	0.909 (0.907, 0.910)	37.874 (37.766, 37.982)	0.416 (0.416, 0.416)
	3D Total	0.909 (0.908, 0.910)	37.821 (37.772, 37.870)	0.365 (0.353, 0.376)
	Total	0.927 (0.926, 0.928)	38.705 (38.644, 38.766)	0.447 (0.426, 0.469)
GenDSA	2D IA	0.966 (0.964, 0.967)	40.570 (40.248, 40.892)	0.803 (0.802, 0.804)
	2D CVS	0.963 (0.961, 0.966)	40.400 (40.071, 40.729)	0.725 (0.708, 0.743)
	2D AVM	0.960 (0.957, 0.962)	40.239 (39.887, 40.590)	0.704 (0.703, 0.704)
	2D AVF	0.965 (0.963, 0.967)	40.649 (40.314, 40.984)	0.342 (0.255, 0.429)
	2D MMD	0.964 (0.960, 0.967)	40.557 (40.233, 40.882)	0.215 (0.215, 0.215)
	2D Thorax	0.968 (0.966, 0.970)	40.692 (40.386, 40.997)	0.215 (0.215, 0.215)
	2D Total	0.964 (0.963, 0.965)	40.519 (40.385, 40.653)	0.501 (0.459, 0.542)
	3D IA	0.893 (0.891, 0.895)	37.399 (37.291, 37.507)	0.215 (0.215, 0.215)
	3D CVS	0.891 (0.889, 0.892)	37.074 (36.975, 37.173)	0.251 (0.217, 0.285)
	3D AVM	0.887 (0.885, 0.889)	37.085 (36.975, 37.194)	0.456 (0.456, 0.456)
	3D AVF	0.894 (0.892, 0.895)	37.368 (37.261, 37.476)	0.456 (0.456, 0.456)
	3D MMD	0.890 (0.889, 0.892)	37.167 (37.059, 37.275)	0.456 (0.456, 0.456)
	3D Total	0.891 (0.890, 0.892)	37.218 (37.171, 37.266)	0.369 (0.349, 0.388)
	Total	0.915 (0.914, 0.916)	38.313 (38.252, 38.374)	0.440 (0.415, 0.465)
SAVE-Net	2D IA	0.975 (0.974, 0.976)	42.373 (42.074, 42.672)	0.382 (0.382, 0.383)
	2D CVS	0.974 (0.973, 0.976)	42.378 (42.046, 42.709)	0.350 (0.342, 0.357)
	2D AVM	0.969 (0.966, 0.971)	41.613 (41.272, 41.954)	0.341 (0.340, 0.341)
	2D AVF	0.970 (0.968, 0.971)	41.402 (41.077, 41.726)	0.164 (0.122, 0.207)
	2D MMD	0.964 (0.961, 0.967)	40.853 (40.481, 41.225)	0.102 (0.102, 0.102)
	2D Thorax	0.971 (0.969, 0.973)	41.742 (41.411, 42.074)	0.102 (0.102, 0.102)
	2D Total	0.970 (0.970, 0.971)	41.727 (41.590, 41.864)	0.240 (0.220, 0.260)
	3D IA	0.942 (0.941, 0.943)	40.477 (40.361, 40.593)	0.102 (0.102, 0.102)
	3D CVS	0.944 (0.942, 0.945)	40.513 (40.400, 40.626)	0.120 (0.103, 0.138)
	3D AVM	0.942 (0.941, 0.944)	40.535 (40.416, 40.653)	0.223 (0.223, 0.223)
	3D AVF	0.941 (0.940, 0.942)	40.396 (40.283, 40.508)	0.224 (0.223, 0.224)
	3D MMD	0.947 (0.945, 0.948)	40.947 (40.808, 41.085)	0.224 (0.224, 0.224)
	3D Total	0.943 (0.943, 0.944)	40.573 (40.519, 40.628)	0.180 (0.170, 0.189)
	Total	0.951 (0.948, 0.956)	40.764 (40.673, 40.798)	0.212 (0.200, 0.224)

Table S6. The results of descriptive statistical results for overall image quality

Categories	Real	Generating
2D IA	4.951 ± 0.215	4.941 ± 0.235
2D CVS	4.867 ± 0.340	4.847 ± 0.360
2D AVM	4.941 ± 0.235	4.929 ± 0.256
2D AVF	5.000 ± 0.000	4.950 ± 0.218
2D MMD	5.000 ± 0.000	4.900 ± 0.300
2D Total	4.930 ± 0.255	4.910 ± 0.286
3D IA	4.975 ± 0.156	4.960 ± 0.196
3D CVS	4.929 ± 0.258	4.892 ± 0.269
3D AVM	5.000 ± 0.000	4.986 ± 0.119
3D AVF	4.917 ± 0.276	4.867 ± 0.340
3D MMD	4.833 ± 0.373	4.733 ± 0.512
3D Total	4.950 ± 0.218	4.928 ± 0.266
Total	4.940 ± 0.237	4.919 ± 0.276

Table S7. Inter-observer consistency analysis results of overall image quality

Categories	Real K	Generating K
2D IA	0.681*	0.636*
2D CVS	0.882*	0.885*
2D AVM	1.000*	0.736*
2D AVF	0.777*	0.454*
2D MMD	0.688*	0.762*
3D IA	0.932*	0.736*
3D CVS	1.000*	0.767*
3D AVM	1.000*	0.83*
3D AVF	0.869*	0.596*
3D MMD	1.000*	0.551*

$^{*}P < 0.001$.

Table S8. P values of the Wilcoxon tests for overall image quality

Categories	R1	R2	R3	R4	R5
2D IA	0.157	0.157	0.059	0.083	0.083
2D CVS	0.157	0.083	0.317	0.083	0.157
2D AVM	0.083	0.157	0.317	0.157	0.083
2D AVF	0.317	0.317	0.157	0.317	1.000
2D MMD	0.317	0.317	1.000	1.000	1.000
3D IA	0.157	0.083	0.083	0.317	0.157
3D CVS	0.083	0.083	0.157	0.083	0.157
3D AVM	0.317	0.157	0.157	0.317	0.317
3D AVF	0.083	0.317	0.157	0.157	0.157
3D MMD	1.000	0.317	0.317	0.317	1.000

Table S9. The results of descriptive statistical results for diagnostic confidence

Categories	Real	Generating
2D IA	4.912 ± 0.283	4.859 ± 0.401
2D CVS	4.900 ± 0.300	4.853 ± 0.372
2D AVM	4.941 ± 0.235	4.812 ± 0.473
2D AVF	4.900 ± 0.300	4.775 ± 0.418
2D MMD	4.800 ± 0.400	4.700 ± 0.458
2D Total	4.908 ± 0.289	4.836 ± 0.411
3D IA	4.900 ± 0.300	4.865 ± 0.342
3D CVS	4.943 ± 0.287	4.857 ± 0.407
3D AVM	4.943 ± 0.232	4.857 ± 0.350
3D AVF	4.867 ± 0.340	4.733 ± 0.442
3D MMD	4.867 ± 0.340	4.733 ± 0.442
3D Total	4.912 ± 0.297	4.840 ± 0.383
Total	4.910 ± 0.293	4.838 ± 0.397

Table S10. Inter-observer consistency analysis results of diagnostic confidence

Categories	Real K	Generating K
2D IA	0.756*	0.843*
2D CVS	1.000*	0.890*
2D AVM	1.000*	0.736*
2D AVF	0.722*	0.642*
2D MMD	0.688*	0.762*
3D IA	0.889*	0.893*
3D CVS	0.569*	0.737*
3D AVM	0.735*	1.000*
3D AVF	0.712*	0.787*
3D MMD	0.712*	0.720*

$^{*}P < 0.001$.

Table S11. P values of the Wilcoxon tests for diagnostic confidence

Categories	R1	R2	R3	R4	R5
2D IA	0.157	1.000	0.083	0.083	0.083
2D CVS	0.317	0.157	0.317	0.157	0.317
2D AVM	0.083	0.157	0.317	0.157	0.083
2D AVF	0.317	1.000	0.157	0.157	1.000
2D MMD	0.317	0.317	1.000	1.000	1.000
3D IA	0.317	0.157	0.083	0.317	1.000
3D CVS	0.083	0.083	0.157	0.157	0.157
3D AVM	0.317	0.317	0.317	0.317	0.317
3D AVF	0.157	0.157	0.317	0.157	0.317
3D MMD	1.000	1.000	0.157	0.157	1.000

REFERENCES

Su, J., Ahmed, M., Lu, Y., Pan, S., Bo, W., and Liu, Y. (2024). Roformer: Enhanced transformer with rotary position embedding. Neurocomputing 568, 127063

4.2 Figures

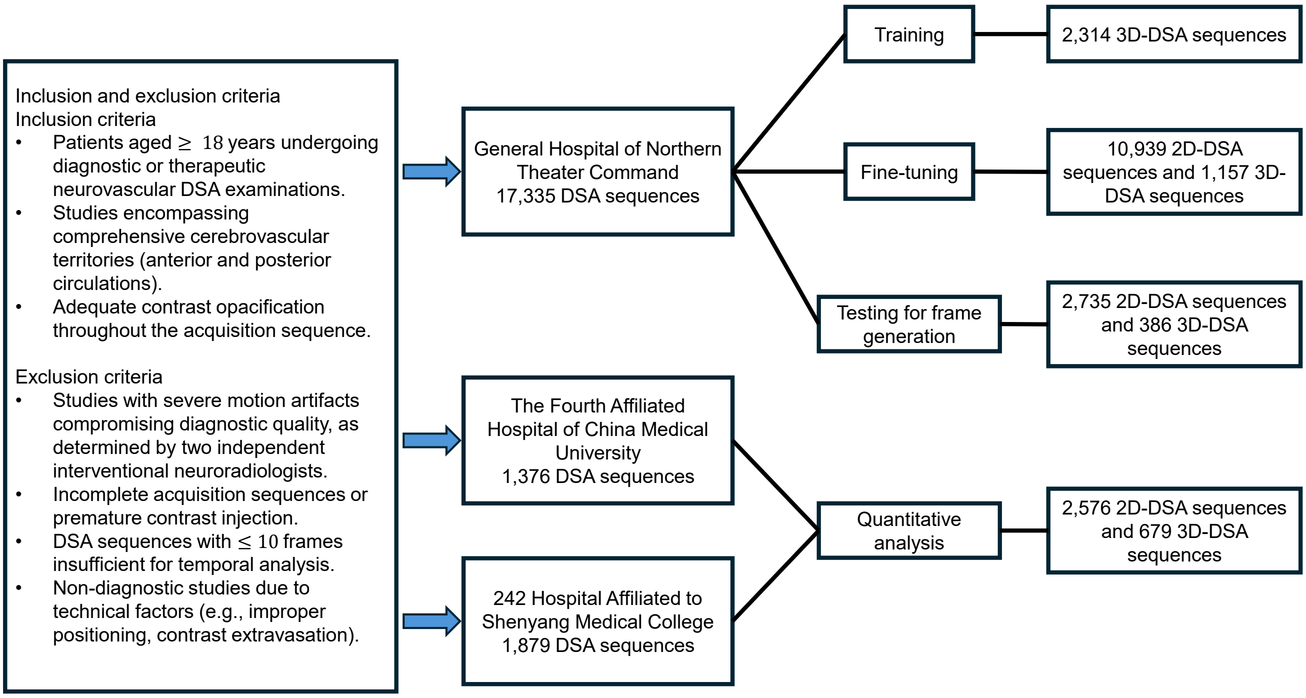

Figure S1. Flowchart illustrating the inclusion and exclusion criteria, followed by data collection and division.

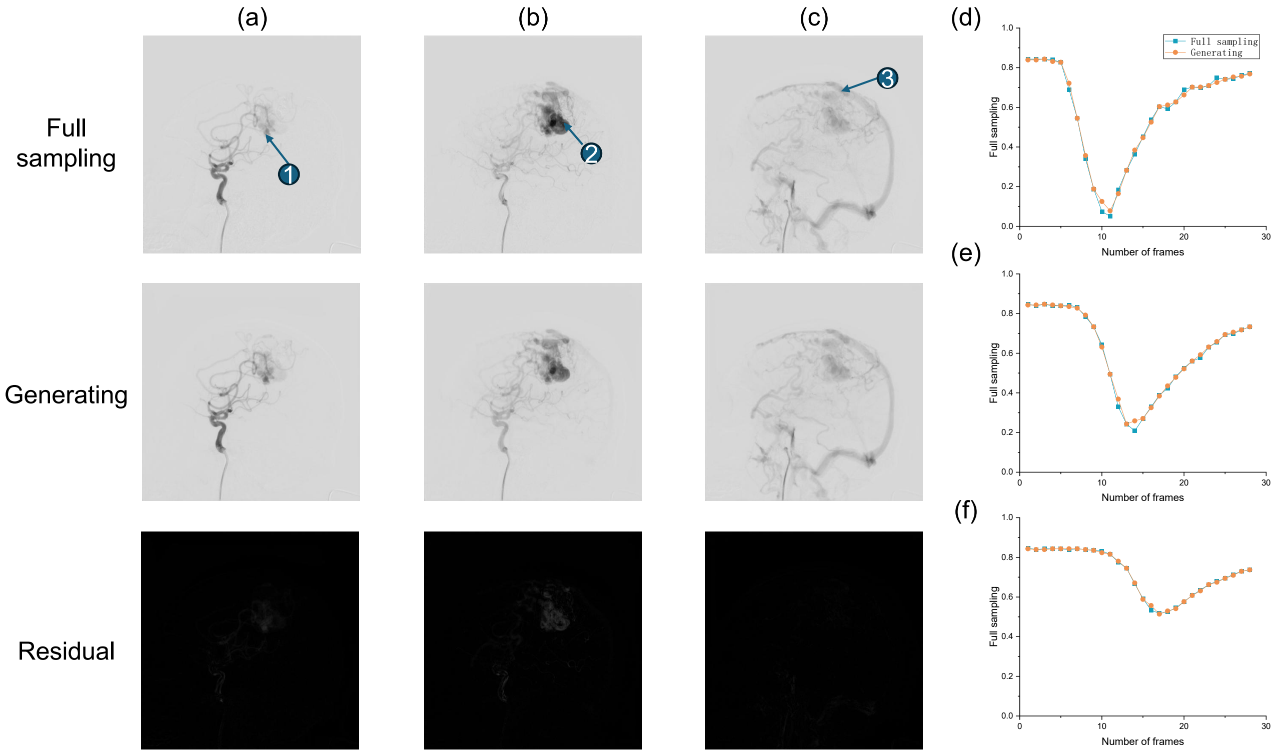

Figure S2. Generative capabilities of SAVE-Net for AV-Shunts. (A) Generated results of the arterial phase. (B) Generated results of the AV-Shunts phase. (C) Generated results of the venous phase. (D) The time-intensity curves corresponding to point 1. (E) The time-intensity curves corresponding to point 2. F The time-intensity curves corresponding to point 3.

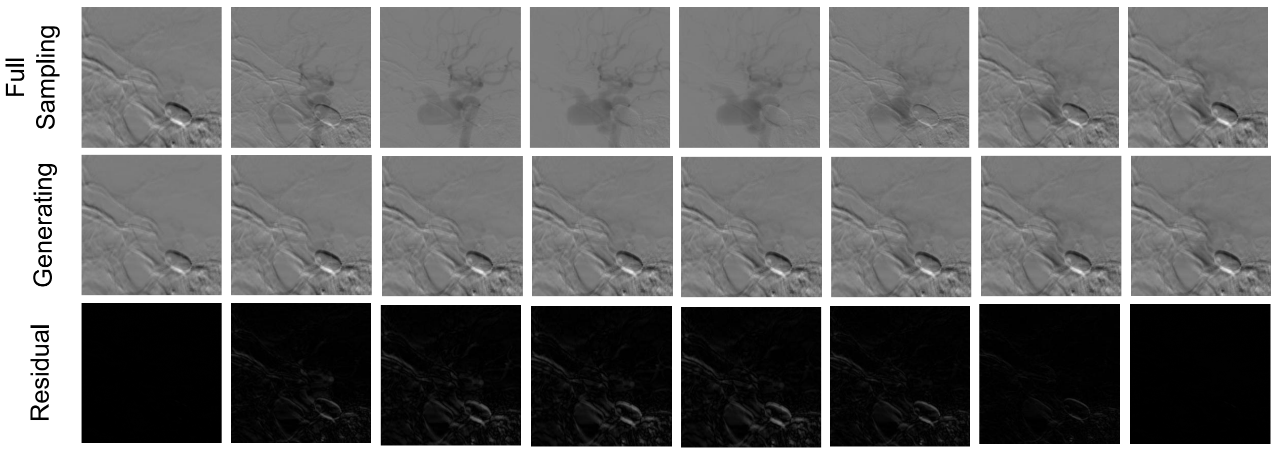

Figure S3. Results in 2D-DSA mode with severe artifacts in the input frames. The images from left to right are consecutive frames of the same sequence, where the first frame and the last frame are the input frames of the model.

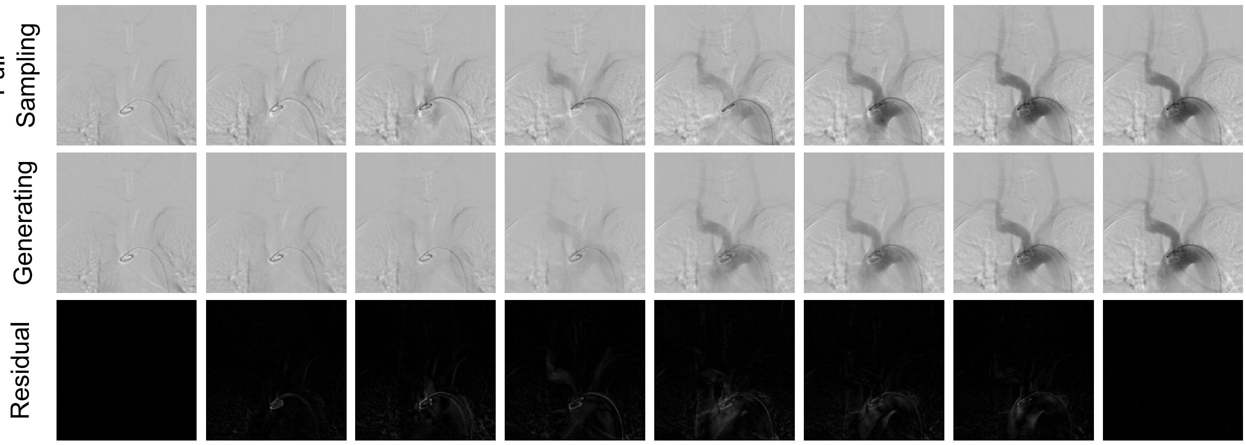

Figure S4. Results under large differences between the first and last frames in 2D-DSA mode. The images from left to right are consecutive frames of the same sequence, where the first frame and the last frame are the input frames of the model.

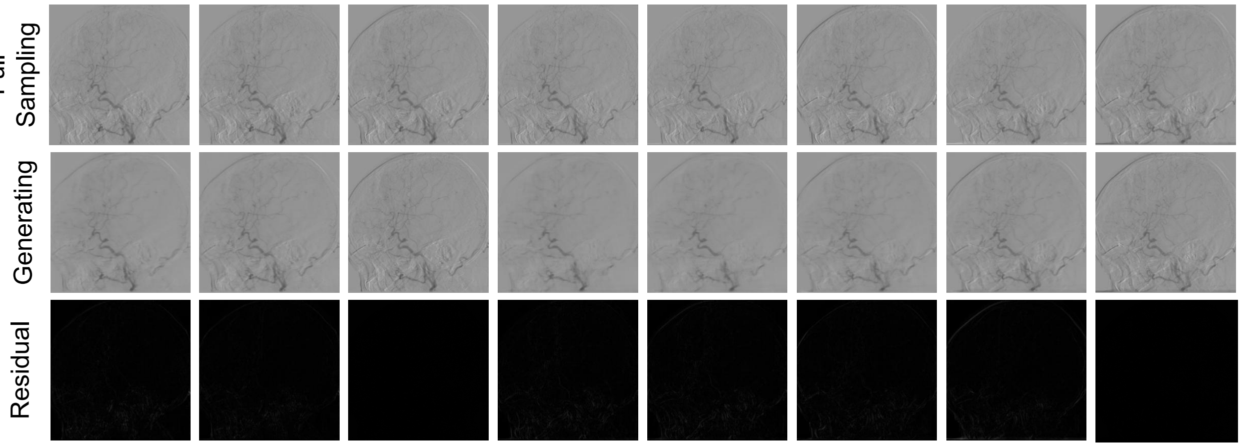

Figure S5. Results in 3D-DSA mode with unclear vascular structures. The images from left to right are consecutive frames of the same sequence, where the first frame and the last frame are the input frames of the model.
